# Synergistic N‑Vacancy and S‑Scheme Engineered CaF_2_/g‑C_3_N_4_ Heterojunction for Photocatalytic Aromatic Phenol Degradation: DFT, Phytotoxicity, and Environmental Impact

**DOI:** 10.1021/acsomega.6c06038

**Published:** 2026-09-09

**Authors:** Dwaipayan Dhar, Sonali Sengupta

**Affiliations:** Department of Chemical Engineering, 30133 Indian Institute of Technology Kharagpur, Kharagpur 721302, India

## Abstract

Advanced oxidation technology using photocatalysis is widely regarded as a promising strategy for removing pollutants from wastewater. However, developing efficient visible-light-driven photocatalysts for practical applications remains a significant challenge. In this study, a novel, recyclable S-scheme heterojunction, g-C_3_N_4_/CaF_2_ (GCN/CF), was successfully synthesized via a hydrothermal method. The unique charge-transfer pathway in GCN/CF facilitates enhanced charge separation while retaining the robust redox properties of the composite materials. UV, PL, and TRPL analyses confirmed the generation of an increased number of photoinduced carriers. The GCN/CF composite exhibited significantly improved degradation rates, with kinetic constants 10 times higher than those of the no-catalyst system and 4 times greater than those of pure GCN. Remarkably, 40-GCN/CF achieved degradation efficiencies of 69–74%, 46–56%, and 40% for pollutants in tap water, pond water, and Hooghly River water, respectively. The introduction of nitrogen vacancies improved charge-carrier mobility and promoted oxygen adsorption at N-vacancy sites, as supported by experimental results. Furthermore, the generation of reactive species such as O_2_^–^• and OH• played a critical role in pollutant degradation. LC-MS analyses identified intermediate products and degradation pathways. This study provides valuable insights for designing advanced S-scheme photocatalysts for real wastewater treatment applications.

## Graphical abstract

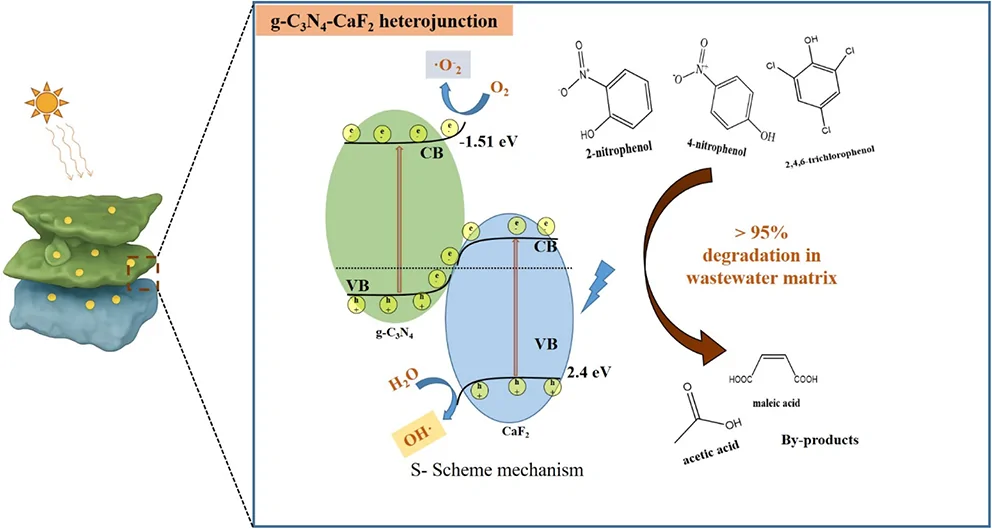

## 1 Introduction

Nitrophenols belong to a group of chemical compounds that are listed among the leading 114 toxic substances by the Environmental Protection Agency (EPA).^1^ Agrochemical factories, defense industries, pharmaceuticals, refineries, pesticides, herbicides, and wood preservatives use aromatic nitrophenols (NPs) as raw materials.^2,3,4^ Residual NPs are released into the environment, especially in wastewater. They can accumulate in animals and plants, damaging blood and nervous systems irreversibly. Due to the nitro group’s strong electron affinity (−NO_2_), 4-nitrophenol is stable and toxic, making it unaffected by biochemical treatment.^5^ These hazardous aromatic phenols in industrial and agricultural wastewater have been treated using adsorption, photocatalysis, electrolytic processes, reduction, and more.^6,7,8^

Semiconductor photocatalysis represents an effective and environmentally friendly approach for addressing environmental concerns.^9,10^ It involves harnessing solar energy to degrade pollutants^11^ and generate hydrogen^12^ or produce solar fuels, utilizing excess CO_2_ as a raw material.^13^ Graphitic carbon nitride (g-C_3_N_4_, GCN), a carbon-derived nanomaterial, has garnered research attention due to its affordability, resilience, and ease of fabrication.^14,15^ GCN is remarkable as a metal-free π-conjugated photocatalyst. It absorbs visible light up to 450 nm due to its well-aligned conduction band (CB) and valence band (VB), generating highly oxidative species. Low surface area and a high charge–carrier recombination rate limit its photocatalytic efficiency.^16,17^ The practical and efficient use of photocatalysis in pollutant degradation depends on reactant adsorption on the photocatalyst surface and effective separation from the reaction mixture.^18,19^ As a result, the combination of a GCN photocatalyst with mesoporous materials or metal-based substances can augment its adsorption characteristics.^20^ A negative CB potential of −1.5 eV for GCN makes it an effective charge carrier when combined with a semiconductor with a deep VB and strong redox capabilities.

This work uses calcium fluoride (CaF_2_, CF), a novel Ca-containing semiconductor, to achieve a remarkable photocatalytic performance. UV-light-photoactive CF is a p-type semiconductor.^21^ Combining CF and GCN to make a composite photocatalyst is promising. This is explained by its deep valence band (2.4 eV), photochemical stability, abundance, and environmental friendliness. The incorporation of structural defects, such as surface nitrogen (N) vacancies in the GCN structure, has been adopted as an effective method for significantly enhancing photocatalytic activity.^22,23^ The vacancies created in GCN serve as additional reactive sites, leading to more efficient catalysis of surface photoreactions.^24,25^ Furthermore, the creation of vacancies can significantly impact the crystallinity of GCN, thereby exerting a substantial influence on its photocatalytic performance.^26,27^ Semiconductor engineers use p–n junctions to improve carrier transport kinetics and performance. P-type (GCN) and n-type (CF) semiconductors have very different electronic conductivities, so free carrier diffusion creates a potential difference on either side of the junction.^28^ A gradient in the carrier concentration in physical space creates a robust internal electric field (IEF) across the junction. This IEF guides the dynamic vectorial transfer of charge carriers.^29^ Given this, synergistically combining both materials and developing heterojunctions could increase the driving force of the IEF at the GCN/CF heterojunction, which is an unexplored area.

Encouraged by this goal, this study synthesizes CF heterojunctions anchored to N-vacancy-rich GCNs. Solar light luminescence was used to photoreduce 2-nitrophenol (2-NP), 4-nitrophenol (4-NP), and 2,4,6-trichlorophenol (TCP). This composite was synthesized, characterized, and used to degrade phenols for the first time. Comprehensive analyses showed that CF anchoring affected the composite’s photodegradation yield. Reaction parameters, such as pH, ions, scavengers, etc., were examined. The heterojunctions were used to remove phenolic compounds from real wastewater.

## 2 Results and Discussion

### 2.1 Structural and Compositional Analysis

To examine sample crystal structure, phase purity, and crystallinity, X-ray diffraction was performed. Figure 1 shows the diffraction patterns of individual GCN, CF, and GCN/CF composites. Observations of bare GCN revealed two distinct peaks at 2θ = 13° and 27.2°, indexed as the (100) and (002) facets, respectively, using JCPDS card no. 87-1526.^30,31,32^ These peaks are two graphite phases of carbon nitride. The tiny-intensity peak at 2θ = 13° indicates interlayer packing. The interplanar stacking arrangement inside the carbon nitride framework of the aromatic system is represented by the stronger peak of (002) facet.^33^ Pure CF powder diffraction was compared to ICDD 2011 PDF 04-007-5933. Diffraction peaks at 2θ = 28.6°, 48.4°, 57.4°, and 68.9°, indexed as (111), (220), (311), and (400) facets, respectively, were observed. These indexed facets can be attributed to a fluorite -like structure with a space group of *Fm* ^*¯*^ *3m*. Composite structures of GCN/CF have all the clear facets of their bare-type phase diffraction patterns except the weak (100) GCN facet. The high crystallinity of CF nanoparticles dominates the weak crystal facets, and their highly crystalline structure likely chemically combines well with the GCN phase crystals. The composite diffraction data show a slight left shift of the GCN (002) facet. The d-spacing (d) value of the plane increases as the 2θ value of the (002) facet decreases. This shows that CF nanoparticles correctly intercalated inside the interplanar stacking arrangement of GCN nanosheets. Increasing CF weight raised the d value. As the CF dosage changes , the peak areas of the (111) and (220) facets change, demonstrating crystallinity and lattice constant value alterations (a, b, c).

**Figure 1:**
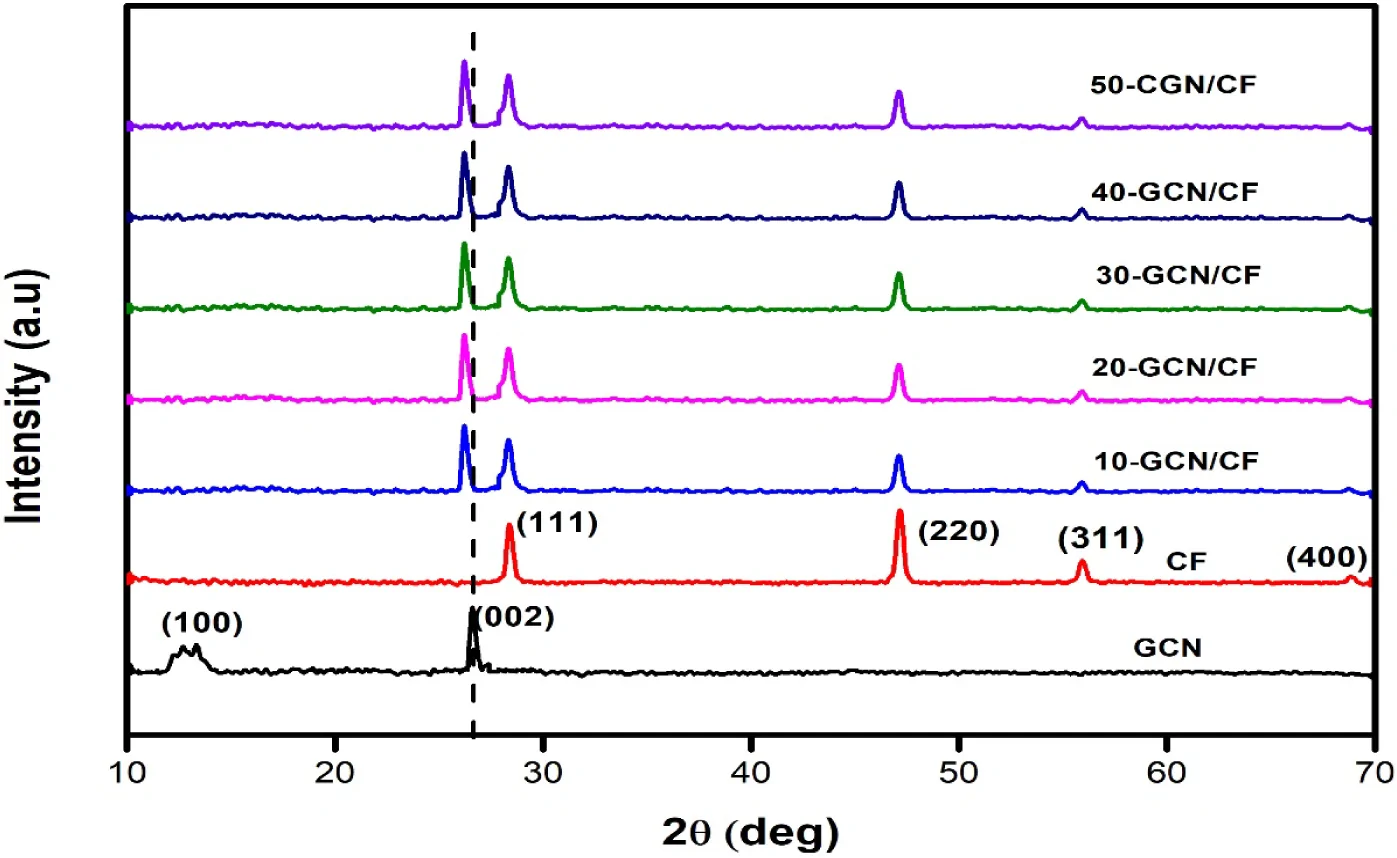
XRD patterns of GCN, CF, and different GCN/CF heterojunctions.

The crystal size can be calculated from the XRD data. The lattice constant values of the simple cubic structure of CF were calculated by the phase equation for the cubic unit cell:

1 $$\frac{1}{d^{2}}=\left(h^{2}+k^{2}+l^{2}\right)\left(\frac{1}{a^{2}}\right)$$

The value of the lattice constant, a = b = c = 5.46 Å, is in good agreement with the previously reported data.^34^ The value of the lattice constant changed with the varied ratio of GCN:CF for different GCN/CF heterojunctions, as the value of d-spacing changed for peak shifting. The crystal sizes of the bare and heterostructured catalyst nanoparticles were determined by the equation:

2 $$size\;of\;crystal\left(D\right)=\frac{k\lambda }{\beta \;\cos\theta }$$

where *D* is the size of the crystal, *k* is the shape factor, θ is the Bragg angle, and β is the full width at half maximum (FWHM). The crystal sizes are represented in Table S1, ESI.

For each as-prepared sample, the BET surface area and Barrett–Joyner–Halenda (BJH) pore size distribution were measured. The mesoporous catalyst samples have a type IV-like N_2_ adsorption-desorption isotherm with a hysteresis loop (Figure S1 a, ESI). BET analysis showed an increase in the specific surface area of the heterostructure as the CF percentage increased. Pure GCN had the lowest specific surface area, resulting in poor pollutant adsorption and degradation. The surface area increased with CF, and 40-GCN/CF exhibited the highest specific surface area. For 50-GCN/CF, the surface area declined sharply. CF nanoparticle accumulation on GCN sheets inhibits surface area growth. BET surface area and BJH pore-size distribution analyses showed analogous outcomes (Figure S1 b, ESI). Bulk GCN and pure CF have low catalytic activity due to their small pores. However, the 40-GCN/CF sample displayed the largest pore size, indicating the finest surface properties among the as-prepared samples. Excess CF blocks heterostructure pores, reducing pore volume and catalytic properties. The calculated values of the different surface areas and pore diameters are elaborated in Table 1.

**Table 1:** BET Analysis Parameters

Sample	Specific surface area (m^2^/g)	Pore diameter (Ǻ)	Pore volume (cm^3^/g)
GCN	16.57	13.34	0.37
CF	25.99	16.31	0.605
10-GCN/CF	39.22	16.93	0.681
20-GCN/CF	46.73	17.86	0.714
30-GCN/CF	51.38	17.91	0.773
40-GCN/CF	76.79	19.23	0.791
50-GCN/CF	56.97	15.83	0.697

FTIR detected chemical functional groups in bare GCN, CF, and all GCN/CF composites. The breathing mode of the tris-triazine ring structure of carbon nitride is indicated by the peak at 812 cm^–1^ in bare GCN^35,36^ (Figure S2, ESI). The peaks between 1200 and 1600 cm^–1^ (1230, 1329, 1570, and 1640 cm^–1^) correspond to heterocyclic group C–N bond stretching vibration.^37^ The composite structures show these characteristic peaks. The Ca–F bond manifests as a minor stretching at 450 cm^–1^ in bare CF samples. All distinct stretching vibrations were present in GCN/CF composite structures, confirming coupling and heterojunction formation.

### 2.2 Morphology Analysis

Figure 2 displays FESEM images of bare GCN, CF, and heterojunctions of different GCN/CF composites. Figure 2a shows multiple sheet-like architecture of bulk GCN with a smooth upper surface. The bare CF nanoparticles appeared hexagonal and asymmetrical (Figure 2b). According to the FESEM images, the CF nanoparticles were 80–130 nm in size. The ternary composite structure roughened GCN’s smooth surface and wrapped CF nanoparticles around the GCN sheets. Figure 2c–f depicts different % CF on GCN heterojunctions. This shows a strong interaction between bare GCN and CF nanoparticles. Perfect CF particle dispersion into GCN units boosts catalyst surface area and photocatalytic activity.

**Figure 2:**
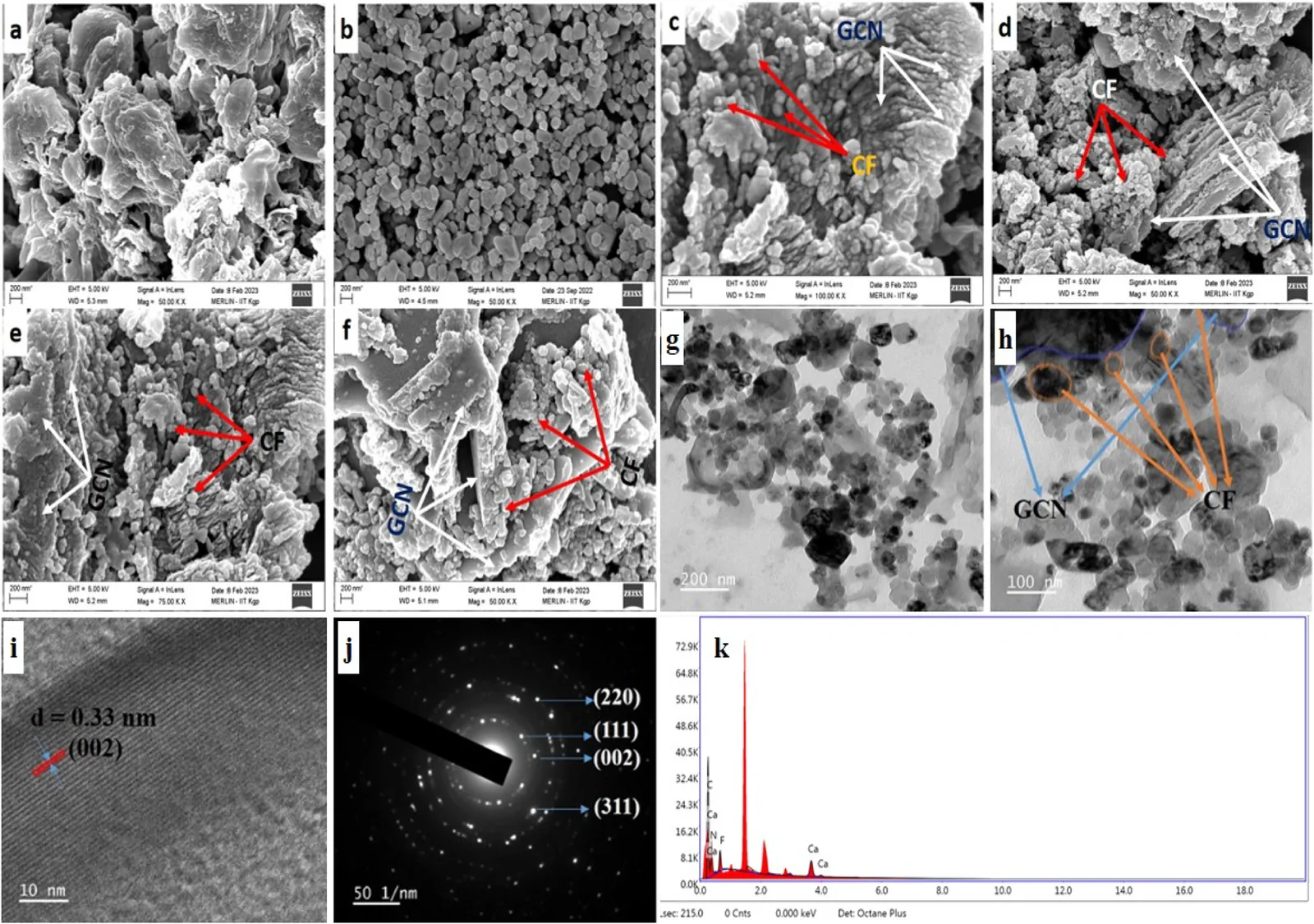
FESEM images of (a) bare GCN, (b) bare CF, (c) 10-GCN/CF, (d) 20-GCN/CF, (e) 30-GCN-CF, and (f) 40-GCN/CF; HRTEM images of (g, h) 40-GCN/CF; (i) lattice fringes; (j) SAED pattern; (k) EDX elemental analysis of C, N, Ca, and F.

The surface morphology and combination were examined using high-resolution transmission electron microscopy (HRTEM) (Figure 2g–j). TEM images showed that CF nanoparticles adhered evenly to GCN sheets (Figure 2 g and h). No agglomerated structures were observed at 40% CF loading on GCN, which improved charge-transfer kinetics. HRTEM images of the lattice fringe (Figure 2i) show a 0.33 nm interlayer d-spacing due to the (002) facet of GCN sheets. This indicates higher crystallinity in the 40-GCN/CF heterojunction. The selected-area electron diffraction (SAED) pattern shows several bright spots in the inverse lattice plane (Figure 2j) indexed by (002) facet of GCN and (220), (111), and (311) facets of CF. These are in juxtaposition to the XRD results. This signifies the successful formation of the 40-GCN/CF heterojunction, which possesses a polycrystalline feature. Energy-dispersive X-ray (EDX) spectroscopy was used to analyze the as-prepared catalyst samples (Figure 2 k). The elemental mapping identified C, N, Ca, and F particles (Figure S3, ESI). The catalyst mapping displays a uniform distribution of sample atoms. Table S2, ESI, illustrates the constituent element percentages.

### 2.3 Analysis of Chemical State

Chemical constituents and their valence states in pure and heterojunction compounds were investigated by XPS. Survey spectra of pure GCN revealed C 1s and N 1s peaks at 285.6 and 400.2 eV, respectively (Figure 3 a). Conversely, pure CF survey spectra showed Ca 2p and F 1s. Figure 3b shows the basic constituent materials of the GCN/CF heterostructure, and no impurities or foreign materials in the spectra are observed. High sample purity helps prolong catalytic activity and prevent catalyst poisoning. Figure 3c shows deconvoluted high-resolution C 1s spectra of pure GCN and the heterojunction sample (40-GCN/CF). Two distinct peaks at 284.36 and 288.58 eV were observed in pure GCN, presumably due to the presence of advantageous carbon particles on the surface of bulk GCN due to air exposure and the sp^2^ hybrid state of the N=C–N functional group in the s-triazine ring.^38^ The heterostructure showed a slight shift of the C 1s peak associated with the sp^2^ s-triazine ring. The C 1s spectrum of the GCN/CF composite exhibits an additional component at approximately 286.1 eV, indicating a modified carbon chemical environment resulting from interfacial interaction with CaF_2_. Consequently, the peak is regarded as an indication of electronic disturbance during composite formation, which replaced the nitrogen atom in the N=C–N group. After replacing the N atom, a nitrogen vacancy is formed, which traps electrons during charge transfer under visible light and reduces recombination. The N 1s high-resolution spectrum in the pure GCN sample showed three deconvoluted peaks (Figure 3d) at 399.6, 401.2, and 404.3 eV, which indicate the pyridinic sp^2^ two-coordinated N atom present in the N=C–N s-triazine ring (N1),^39^ three-coordinated pyrrolic N atom interlinked with sp^3^ H–N–C_3_ (N2)^40^ and graphitic (C–NH−)_*x*_ group, respectively. Interestingly, a slight left shift of the N1 peak corresponding to 399.6 eV was observed as a result of insertion of sp^2^ N-atom by Ca atoms into the GCN skeleton, which was previously verified by the C 1s spectra shifting. Notably, a sharp decrease in the peak area corresponding to the N2 high-resolution peak at 401.2 eV was observed in the GCN/CF heterojunction compared to the pure one. The N-atom substitution from the N=C–N s-triazine ring and sp^3^ H–N–C_3_ (N2) framework created a significant number of N-vacancy defects for which the electron density significantly increased and the C–Ca bond formed. Elemental analysis from the XPS peak areas (Table 2] showed a significant decrease in N species, indicating an N-vacancy-rich GCN/CF heterojunction.

**Figure 3:**
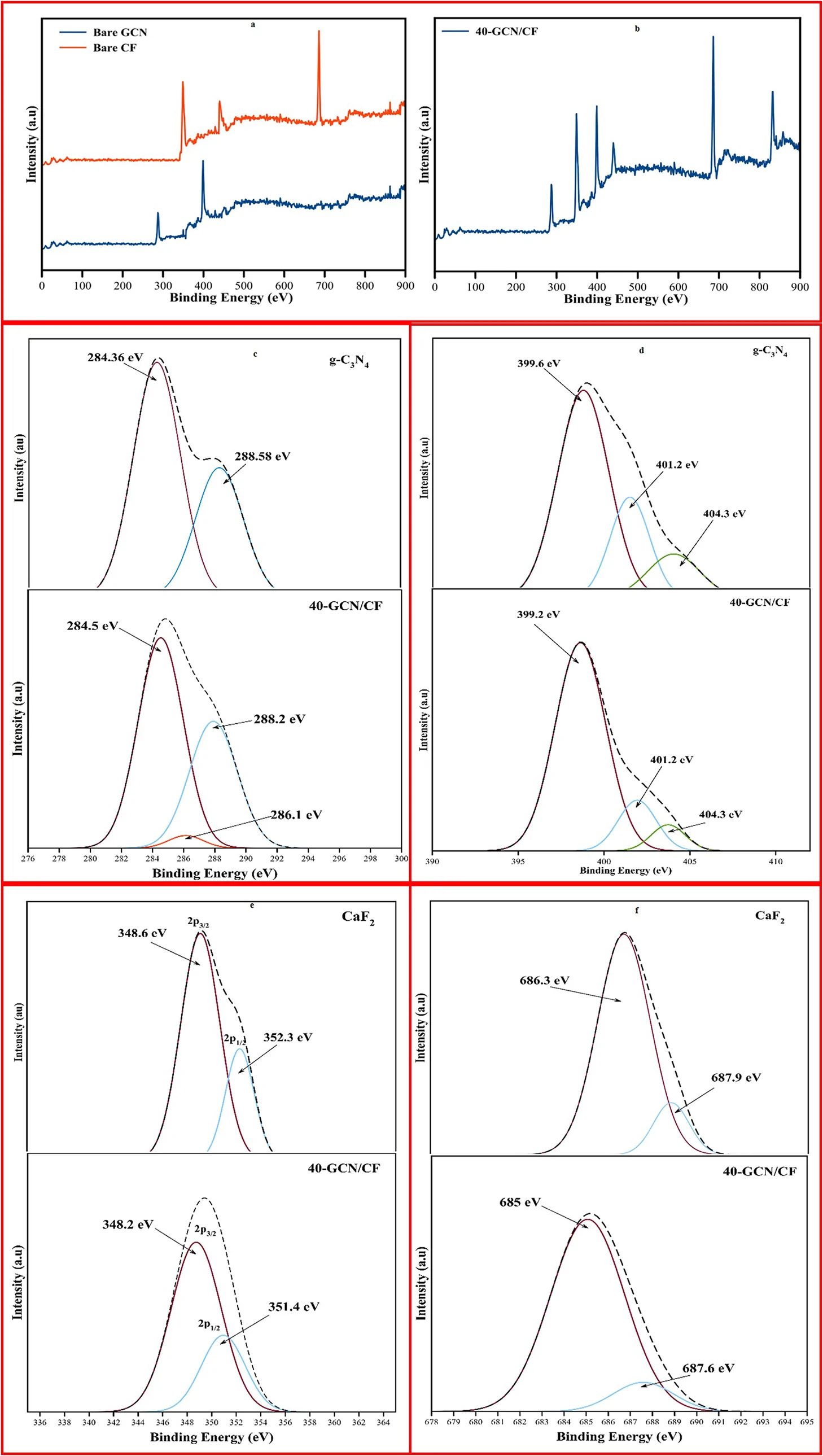
XPS spectra of differently prepared samples: (a) survey spectra of pure GCN and CF; (b) survey spectra of the 40-GCN/CF heterojunction; high-resolution spectra of (c) C 1s, (d) N 1s, (e) Ca 2p, and (f) F 1s.

**Table 2:** Elemental Analysis from XPS Peak Areas (N_2_)

Sample	% Area under N1 curve	% Area under N2 curve
Pure GCN	67.81	47.92
GCN/CF	59.93	42.13

The increased N-vacancy data from Table 2 were also verified by the increased C/N ratio from pure GCN to the GCN/CF heterojunction. The elemental profiles in the XPS spectra showed an increase in the C/N ratio for both N1 and N2 peaks [Table 3].

**Table 3:** C/N Percentage Calculation from C 1s and N 1s XPS Peak Areas

Sample	% of C	C/N ratio in N1	C/N ratio in N2
Pure GCN	36.87	0.467	0.660
GCN/CF	33.01	0.528	0.751

The high-resolution Ca 2p spectra of the pure CF sample showed two spin-orbit peaks at 348.6 and 352.3 eV upon deconvolution. A slight left shift in the GCN/CF peaks indicates successful bonding with the C atom in the N=C–N s-triazine ring of the GCN lattice. The binding energies decreased by −0.4 and −0.9 eV as the electron density of the Ca^2+^ ion decreased due to bonding with the sp^2^ carbon atom. The high-resolution spectra of F 1s showed two deconvoluted spin orbitals at 686.3 and 687.9 eV, and slight shifts of −0.7 and −0.3 eV in binding energy were noticed in the GCN/CF composite, which implied the presence of (−1) oxidation state of the F atom in the GCN/CF heterojunction.

### 2.4 Optical Properties

The optical absorption edges and experimentally calculated band gap (*E_g_*) of all prepared samples were assessed using UV-VIS absorbance spectra and Tauc plots. Figure 4a shows that pure GCN had absorption edges at 430–440 nm and pure CF at 420–430 nm. Increased CF nanoparticle insertion into GCN sheets shifted the absorption edges to higher wavelengths. Near 530 nm, 40-GCN/CF edged. From the sample Tauc plots (Figure 4b), *E_g_* was calculated. *E_g_* was calculated by extrapolating the tangent of each Tauc plot curve to the *X*-axis and measuring the point where it intersected the horizontal axis. Using eq 3, the Tauc plot was generated:^41^

3 $${\left(\alpha h\vartheta \right)}^{\frac{1}{n}}=A\left(h\vartheta -E_{g}\right)$$

**Figure 4:**
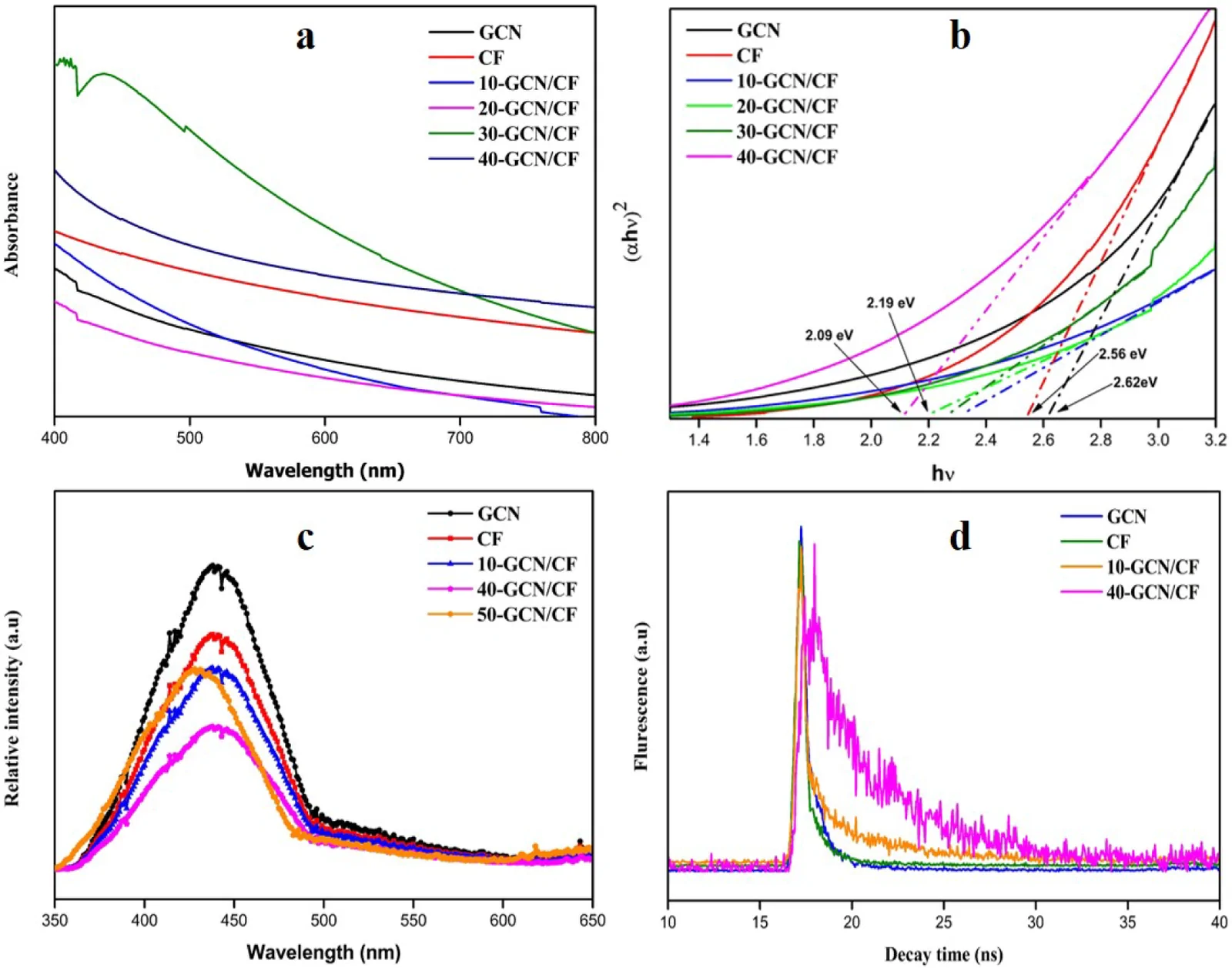
(a) UV-VIS absorbance spectra, (b) Tauc plot, (c) photoluminescence emission pattern, and (d) TRPL decay lifetime of GCN, CF, 10-GCN/CF, 20- GCN/CF, 30- GCN/CF, 40- GCN/CF, and 50- GCN/CF.

where α is the absorbance factor, *h* is the Planck’s constant, ϑ is the frequency, *A* is the proportionality constant, and *E_g_* is the forbidden band gap of a semiconductor sample. The type of semiconductor defines the parameter *n* and it is denoted as 1/2 for direct *E_g_* and 2 for indirect *E_g_*. Since GCN is an indirect band-gap semiconductor material, this *n* value was considered as 2 for these plots. The calculated *E_g_* values for the prepared samples are 2.62, 2.56, 2.31, 2.309, 2.19, and 2.09 eV for pure GCN, CF, 10-GCN/CF, 20-GCN/CF, 30-GCN/CF, and 40-GCN/CF, respectively. In the pure GCN sample, the *E_g_* was much wider, making it unsuitable for visible-light photo activity. The CF sample had an *E_g_* of 2.56 eV. Ca percentage increased stepwise in the heterojunction catalysts, narrowing *E_g_*. The composite exhibits a red-shifted absorption edge and an effective optical gap of 2.09 eV obtained from Tauc analysis. Prior XPS analysis revealed new C–Ca bond formation and N-vacancy defect generation, altering the composites’ optical and electronic structures compared to the pure ones. It can be hypothesized that mixing C and Ca orbitals in the newly prepared samples optimized the band gap. The shifted absorption edges confirmed new electronic states in the GCN/CF samples. Apart from this, the band-edge potentials of both the CB and VB were computed using the following equations:^42^

4.1 $$E_{\mathrm{VB}}=X-E^{c}+0.5E_{g}$$

4.2 $$E_{\mathrm{CB}}=X-E^{c}-0.5E_{g}$$

where X signifies the electronegativity of the significant atoms. The value of X can be determined by the geometric mean of Mullikan electronegativity^43^ of the constituent atoms, and *E* ^c^ signifies the energy of free electrons on the hydrogen scale, which is almost 4.5 eV. The values of the parameters were calculated and are shown in Table S3, ESI. Pristine CaF_2_ is a wide-band-gap fluoride and is not intrinsically active in visible light. The UV–Vis spectrum of the GCN/CF composite shows a red-shifted absorption edge with respect to the parent components. The visible-light response is attributed to the heterojunction structure, interfacial electronic coupling, and defect-related sub-band states rather than the ideal bulk CaF_2_. Furthermore, light scattering from the composite morphology may contribute to the baseline absorption profile. Therefore, the band gap obtained from the Tauc plot should be treated as an effective optical gap of the composite rather than the intrinsic band gap of pristine CaF_2_.

Recombination tendency and photoinduced electron–hole pair separation efficiency are crucial in charge transfer kinetics. Photoluminescence (PL) spectra revealed charge-migration efficiency. Figure 4c shows that pure GCN samples had the highest peak intensity in the PL spectra, followed by CF and 10-GCN/CF samples. For 40-GCN/CF, the lowest intense peak indicates the highest charge-transfer kinetic efficiency and the least charge-carrier recombination. CF doping replaced more C–N bonds in the sp^2^ hybrid state of the N=C–N functional group in the s-triazine ring, making the 40-GCN/CF sample richer in N-vacancy defects. Vacancy defects trapped electrons during recombination, prolonging photocatalysis. Time-resolved photoluminescence (TRPL) decay analysis confirmed charge-carrier lifetime estimates. Charge carriers excited and decayed over time as shown in Figure 4d. The data acquired from Figure 4d decay curve were fitted with a first-order exponential decay model (eq 6) to calculate the average lifetime of charge carrier.

5 $$I\left(t\right)=A\exp\left(-\frac{t}{\tau }\right)$$

where *I* is the carrier-concentration, *A* is the amplitude of the corresponding decay curve, *τ* is the fluorescence lifetime of the sample, and *t* is the time derivative. The fitted data from the exponential decay curves are presented in Table 4.

**Table 4:** TRPL Decay Lifetime of Charge Carriers Calculated from Exponential Decay Fitting Model

Serial no.	Sample	TRPL decay lifetime (ns)
1	GCN	0.85
2	CF	0.91
3	10-GCN/CF	2.73
4	40-GCN/CF	3.707

The 40-GCN/CF samples had the longest decay time (3.707 ns). Recombination is reduced, and charge transfer efficiency is improved in heterojunctions due to more vacancy sites. Pure GCN and CF samples have 0.85 and 0.91 ns lifetimes, respectively, due to high recombination and a wide band gap. GCN sheets containing CF had superior optoelectronic properties and more rapid charge carrier photoexcitation.

### 2.5 Photoelectrochemical Analysis

Photocurrent density responses of each pure and GCN/CF heterojunction sample were recorded under visible-light on–off conditions for evaluating electron–hole separation and charge–carrier transport efficiency. As semiconductor sample charge-generation efficiency increased, the current density increased during the light-on state. The current density dropped to almost 0 mA after switching off the light. This was repeated six times to test photocurrent stability and density. In Figure S4a (ESI), the visible-light current densities of pure GCN, pure CF, and 10-GCN/CF samples were 0.908, 2.627, and 3.701 mA/cm.^2^ The composite of GCN sheets and CF nanoparticles improved charge separation and charge–carrier availability. In Figure S4b (ESI), the maximum photocurrent, 15.317 mA/cm,^2^ was achieved by the 40-GCN/CF heterojunction catalyst, which is 15.7- and 6-fold greater than those of pure GCN and 10-GCN/CF, respectively. This implies that more CF insertion in the GCN framework has increased N-vacancy formation and lowered electron–hole recombination, leading to more carrier separation and longer charge-transfer efficiency. Hence, 40-GCN/CF nanoparticles generated current effectively under visible light.

To study the charge-transfer mechanism, the nature or type of semiconductor composite must be identified. The Mott–Schottky (M-S) plot of pure GCN, CF, and 40-GCN/CF is shown in Figure S4c–e. The conduction type and flat-band potential were calculated from this M-S plot. In Figure S4 c (ESI), pure GCN’s M-S plot has a positive slope, indicating its n-type semiconductivity. CF exhibited a negative slope (Figure S4 d, ESI), confirming its p-type nature. The data obtained from the electrochemical workstation were plotted according to the M-S equation^44^ as mentioned below:

6 $$\frac{1}{C^{2}}=\pm \frac{2}{e\varepsilon \varepsilon_{0}N_{d}}\left(E-E_{fb}-\frac{K_{b}T}{e}\right)$$

where *C* = capacitance as the function of applied potential, *e* = charge of an electron, ε = dielectric constant, ε_0_ = permittivity constant of vacuum, *E_fb_* = flat-band potential, *e* = applied electric potential, *K_b_* = the Boltzmann constant, *T* = room temperature, and *N_d_* = density of dopants.

The plot was linearly fitted with the intercept $\left(\frac{1}{C^{2}}\right)$ equal to 0, and the flat-band potential was calculated as −1.48 eV for GCN and 0.28 eV for CF versus Ag/AgCl reference electrode. The flat-band potentials were converted to the NHE scale with (eq 8),:^45,46,47^

7 $$E_{\mathrm{NHE}}=E_{\frac{\mathrm{Ag}}{\mathrm{Ag}\mathrm{Cl}}}+E_{\mathrm{Ag}/\mathrm{AgCl}}^{0}+0.059\mathrm{pH}$$

The workstation at pH 6 used 0.25 M Na_2_SO_4_ as a potential electrolyte and the reference electrode Ag/AgCl at 0.19 eV. The detailed calculations are explained in the Section S3.3, ESI. GCN and CF had calculated flat-band potentials of −1.21 and 1.9 eV, respectively. The flat-band potential (*E_fb_*) is almost 0.3 eV below for the n-type semiconductor conduction band minimum (CBM) and 0.4 eV above for the p-type semiconductor valence band maximum (VBM). The values of the CBM and VBM of GCN and CF were determined as −1.51 and 2.4 eV, which are in accordance with the UV–Vis absorbance analysis data. Another exciting outcome was observed for the M-S plot of 40-GCN/CF, which showed (Figure S4 e, ESI) a ‘V-shaped’characteristic curve indicating that both the p- and n-type semiconductors formed the heterojunction. Thus, this as-synthesized composite nanocatalyst can be expressed as a p–n heterojunction photocatalyst.

The electrode–electrolyte interface in the workstation leads charge carriers to face charge separation and charge diffusion resistances. Charge carriers face less resistance as the charge generation capacity improves. As-synthesized photocatalyst electrochemical impedance spectrum (EIS) Nyquist plots are shown in Figure. S4f (ESI). Electrical resistance increases with the semicircle radius and area, while charge transfer improves with a smaller EIS arc radius and area. 40-GCN/CF exhibited the lowest resistance among the heterojunction photocatalysts. TRPL and photocurrent density data support these findings.

### 2.6 Photocatalytic Degradation of Different Phenols

As model wastewater pollutants for photocatalytic degradation, 2-NP, 4-NP, and TCP phenolic pollutants were chosen. First, pure GCN, CF, and all heterojunction catalysts were tested for photocatalytic activity by measuring the degradation of 2-NP, 4-NP, and TCP in D.I. water at 15 ppm under visible light (Section S3.1, ESI). As shown in Figure 5a–c, the rate of degradation in the absence of a catalyst is relatively low for all the phenolic compounds (27% for 2-NP, 18% for 4-NP, and 19.3% for TCP), but the rate of degradation was eventually increased as the catalysts were added into the reaction medium. For pristine GCN, the degradation rates were 51.2%, 39.72%, and 43.81% for 2-NP, 4-NP, and TCP, respectively. Even after prolonged light exposure, the degradation rate was low because of the wide band gap and charge–carrier recombination. Incorporating CF into the GCN framework led to a significant increase in the degradation percentage for the heterojunctions. UV–vis spectrum analysis was used to measure pollutant concentrations, showing decreases in NP and TCP percentages in the water solution (FigureS5, ESI). The 40-GCN/CF photocatalyst degraded model D.I. water by 99% for 2-NP, 98.74% for 4-NP, and 94.78% for TCP within 3 h of visible light exposure. Increased N-vacancy and the least charge–carrier reintegration due to heterojunction formation increased GCN/CF composite photocatalytic activity.

**Figure 5:**
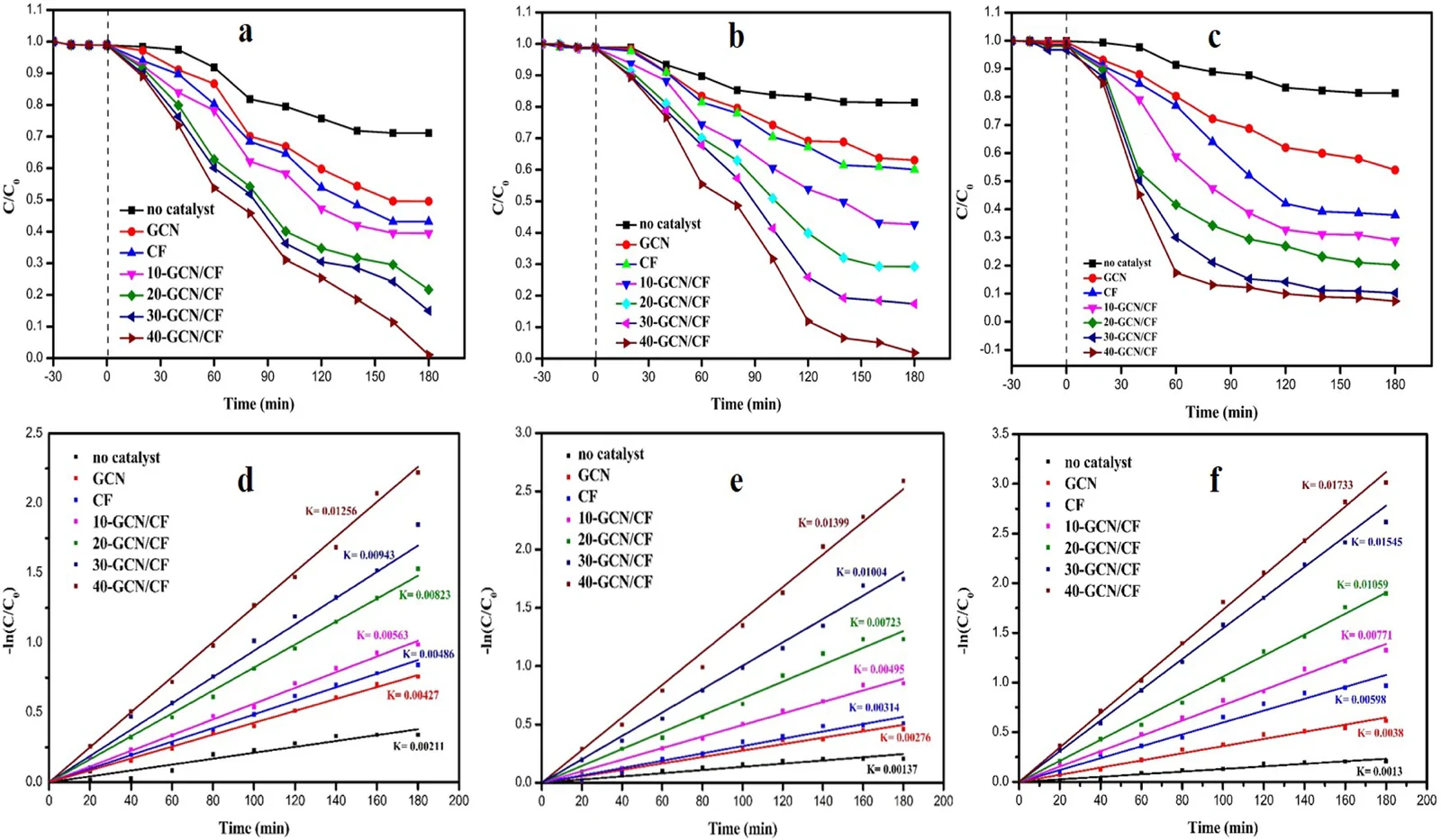
Change in (a) 2-NP, (b) 4-NP, and (c) TCP concentration (C/C_0_) against degradation time (min) and the corresponding kinetic fit of (d) 2-NP, (e) 4-NP, and (f) TCP by as-synthesized catalyst samples.

The photocatalytic degradation of 2-NP, 4-NP, and TCP followed pseudo-first-order reaction kinetics. The kinetic study was carried out by plotting $-\ln\frac{C}{C_{0}}$ versus time. The plots were linearly fitted with a 0 intercept and the slopes of each plot were recorded. This indicates a first-order reaction. In the absence of catalysts, 2-NP, 4-NP, and TCP degradation rate constants (k) were 0.00211, 0.00137, and 0.0013 min^–1^, respectively, while 40-GCN/CF showed rate constants of 0.0125, 0.0134, and 0.0173 min^–1^ for subsequent pollutants, which were 6 to 10 times higher than the no-catalyst run and 3 to 4 times higher than with pristine GCN catalysts. Table S4, ESI, illustrates the rate constants. This study confirmed that the 40-GCN/CF heterojunction is a potential candidate to degrade a wide range of phenolic wastes under visible-light exposure. The high crystallinity, an optimized band structure, higher PL decay lifetimes of charge carriers, and increased N-vacancy are the significant parameters for improving this photocatalytic activity.

### 2.7 Applications in Natural Wastewater and Environmental Impact

As mentioned in Section 2.6, the as-synthesized 40-GCN/CF performed well in model wastewater. In real wastewater samples, this catalyst was tested for the degradation of phenolic waste. To this aim, NPs and TCP dissolved in tap water, local pond water, and Hooghly River water at 15 ppm concentrations were used. The 2-NP, 4-NP, and TCP degradation rates in tap water were found to be 73.26, 69.17, and 70.13%, respectively, while in pond water they were 56.14, 46.91, and 48.78%, respectively. 2-NP, 4-NP, and TCP could be degraded by 39.75, 38.69, and 39.07% with 40-GCN in Hooghly River water. Various kinds of organic pollutants, anions, salts, and silts inhibited efficient catalysis in very turbid Hooghly River water, which was collected from the river several kilometers before its estuary. Solids in suspended and dissolved forms may clog the catalyst’s active sites (Table S5, ESI). 40-GCN/CF performed better in tap and pond water than in Hooghly River water due to lower pollutants and suspended solids. This suggests that 40-GCN/CF can purify lake or municipal tap water from phenolic contaminants satisfactorily.

### 2.8 Influence of Different Reaction Parameters

Parametric studies were carried out on NPs and TCP-dissolved D.I. water under visible light. Simulated wastewater samples were loaded with 10–100 mg/L of 40-GCN/CF to study the catalyst effect. At 15 ppm, 2-NP, 4-NP, and TCP showed the highest degradation percentages after 3 h at catalyst loadings of 60, 78, and 58 mg/L, respectively (Figure 6e); beyond these catalyst concentrations, NP and TCP degradation dropped. The higher catalyst concentration may have prevented photon absorption by the active surface, reducing charge–carrier separation and oxidation.^48^

**Figure 6:**
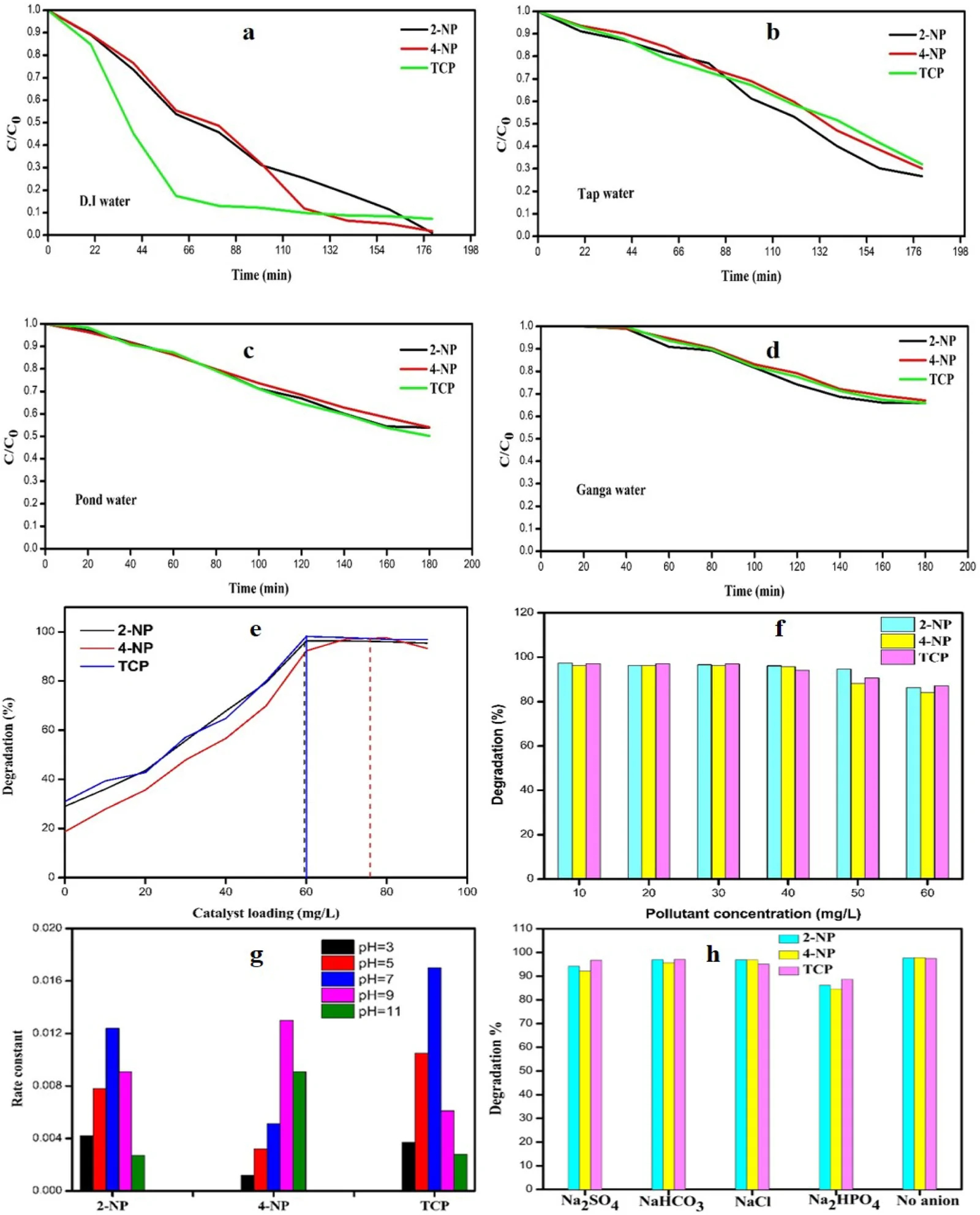
Degradation percentage by 40-GCN/CF in different wastewater matrices: (a)­D.I. water, (b) tap water, (c) pond water, and (d) Hooghly River water [initial concentrations of NPs and TCP in all water samples: 15 ppm]; influence of different reaction parameters: (e) effect of catalyst loading, (f) effect of pollutant concentration, (g) effect of pH, and (h) effect of anions.

The performance of 40-GCN/CF was further tested for different pollutants in wastewater at concentrations between 10 and 60 ppm. The optimum catalyst loadings (60, 78, and 58 mg/L of 40-GCN/CF for 2-NP, 4-NP, and TCP, respectively) were used for these pollutants, and the reactions were carried out for 3 h. For all pollutants, the degradation rate was more than 95%, up to 40 mg/L of the pollutant concentration (Figure 6f). Beyond that, the degradation rate decreased. The mixture degraded over 95% of pollutants at low NP and TCP concentrations due to sufficient photocatalyst and its available active sites. More contaminants may block active sites, reducing light absorption, surface adsorption, and degradation. In this study, NP and TCP concentrations were much higher than those in natural wastewater. This suggests that 40-GCN/CF can effectively remove nitroaromatic pollutants from wastewater.

pH is a key parameter for determining the 40-GCN/CF heterojunction catalyst surface charge and pollutant affinity in different reaction mediums. Figure 6g shows how different pH mediums (acidic and basic) affect the NP and TCP breakdown processes. The pH of the mixtures was initially lowered to almost 3 with 1 M HCl to study GCN/CF efficiency. Adding 1 M NaOH to the reaction mixture examined how basic environment affects catalytic efficiency. The values of the rate constants were observed to be 1.5 times and 2 times greater for 2-NP and TCP at neutral pH, respectively , whereas 4-NP showed the highest breakdown in moderately basic pH conditions (pH = 8–9). The reason behind this observation is that 4-NP is a highly stable compound in aqueous solution and forms hydrogen bonds with water molecules. When the pH increases to 9, it forms a nitrophenolate anion and carries a negative charge in the basic solution.^49,50,51^ High pH attracts negatively charged molecules to the positively charged surface of the catalyst, speeding up the reaction. Degradation decreased when pH reached 11 due to the formation of many negative ions in the medium and the repulsive forces from a higher concentration of anions. At acidic pH, the value of the rate constant was dropped by 4 times of 4-NP due to the repulsion between the catalyst and pollutants. The 2-NP and TCP molecules show pKa values of 7.2 and 6.6, respectively, which implies the fact that these molecules remain in molecular form at a pH lesser than the pKa value. At higher pH, 2-NP and TCP form phenolate anions, which are attracted by the positively charged catalyst surface. At neutral pH, the rate constant was more than 0.012, whereas, at higher pH, it dropped below 0.004. In a highly acidic medium, the availability of OH• is reduced due to high proton generation in the medium, which suppresses the oxidation ability. Apart from these facts, 2-NP is substantially unstable, and TCP experiences a steric hindrance effect. These factors are responsible for their faster degradation at pH = 7. Photocatalytic degradation experiments were repeated in buffer solutions with constant ionic strength to separate pH from ionic strength. The buffer-controlled measurements followed the same pH dependence as the unbuffered experiments, indicating that the protonation or deprotonation of pollutant molecules and the pH-dependent surface charge of the catalyst control degradation rather than uncontrolled ionic strength changes.

Identifying how ionic species affect photocatalytic activity is crucial. Apart from phenolic compounds, agricultural effluents contain metal salts or anionic salts that may poison catalysts or disrupt photocatalytic degradation. After adding anionic metal salts to 15 ppm of NPs and TCP-containing aqueous solution, 40-GCN/CF catalyst degradation reactions showed the effect of anions. In Figure 6 h, the effects of SO_4_^2–^, HCO_3_^–^, Cl^–^, and PO_4_^3–^ were compiled. The study was carried out under ambient temperature (32 °C) at optimum pH, adding 3 mL of 0.05 M anions to the reaction mixture, and the degradation was performed for 3 h. According to Figure 6h, a negligible effect was observed for SO_4_^2–^ and Cl^–^. It is possible that the Cl^–^ ions might have trapped the OH• radicals and converted them to HClO^–^ (eq 9), which does not impede the photocatalytic oxidation.^52,53^

8 $${\mathrm{Cl}}^{-}+\mathrm{OH}•>{\mathrm{HClO}}^{-}$$

9 $${{\mathrm{PO}}_{4}}^{3-}+\mathrm{OH}•>{\mathrm{OH}}^{-}+{{\mathrm{PO}}_{4}}^{2-}$$

10 $${\mathrm{Ca}}^{2+}+{{\mathrm{PO}}_{4}}^{3-}>{\mathrm{Ca}}_{3}{\left({\mathrm{PO}}_{4}\right)}_{2}$$

However, PO_4_^3–^ ions may have lowered catalytic activity, reducing pollutant degradation from 97% to 85%. Eq 9 shows that PO_4_^3–^ traps OH• and converts them to weaker oxidizing species.^54^ Additionally, PO_4_^3–^ may produce metal salts that precipitate on the catalyst surface (eq 11), blocking the active surface area and preventing the active ions approach, hence reducing activity.^55^ Also, hydrolysis of some anionic salts may change the pH of the solution, changing the catalytic surface charge and its repulsion to pollutant molecules. For these reasons, PO_4_^3–^ degrades least among the anions. The anion interference study is a first step in assessing catalyst behavior in ion-containing water, but a more systematic study over a wider ionic-strength range would provide a better understanding of its practical applicability. Each anion concentration ranged from 0.05 to 1.0 M under identical reaction conditions. The degradation efficiency of Cl^–^ and SO_4_^2–^ was only slightly reduced across the concentration range, indicating minimal interference with the photocatalytic process. In contrast, PO_4_^3–^ significantly inhibited degradation efficiency, ranging from 84% at 0.05 M to nearly 50% at 0.1 M and 30.2% at 0.5 M. PO_4_^3–^ induces strong suppression due to competitive adsorption on catalyst surfaces, precipitation, or site-blocking interactions with surface Ca species, reducing access to active sites and inhibiting reactive species generation (Figure S6, ESI). The study shows that 40-GCNCF’s photocatalytic performance is compatible with common anions like Cl^–^ and SO_4_^2–^, but is significantly impacted by phosphate-rich water matrices.

Total organic carbon (TOC) removal efficiencies were found to be 45.3% for Hooghly water, 74.9% for tap water, 63.7% for pond water, and 87.1% for D.I. water with the 40-GCN/CF heterojunction after 3 h of photodegradation (Figure S7, ESI), confirming its outstanding performance in the mineralization of phenols under visible light irradiation.

### 2.9 Phytotoxicity Analysis

Higher concentrations of aromatic phenol-based wastewater can lead to phytotoxic effects in plants because of its diverse constituents. In contrast, treated NP- and TCP-based wastewater actually promoted plant growth and development. This study investigates the impact of photocatalytic degradation on the germination of mung seeds, which typically take about 7 days to sprout (Section S3.2, ESI). Table S6 a, b and c of ESI outlines the germination rate, fresh weight, plant length, and dry weight of mung bean seeds (*Vigna radiata*). The root length and fresh weight were lowest in the raw wastewater, indicating that it had the highest toxicity among all of the samples. The results demonstrated that higher concentrations of phenols in water significantly impeded the root growth. This reduction in shoot and root lengths can occur when roots are exposed to high concentrations of effluent, which adversely affect cell growth. Conversely, seeds flourished in 40-GCN/CF-treated wastewater containing NPs and TCP, suggesting that the toxicity of the pollutant-laden solution was reduced. When compared to seeds germinated in treated tap water and pond water, the germination percentage of seeds sprouted in the untreated phenolic sample appears to be lower. This suggests that the pollutant toxicity was reduced even in tap and pond water. The mung bean bioassay showed that the acute phytotoxicity of the effluent was significantly reduced after photocatalytic treatment, as indicated by improved seed germination, root elongation, and seedling growth relative to the untreated sample. Alongside the final germination percentage, the germination index and root elongation were assessed to quantify the initial seedling response (Figure S8, ESI). The dilution-dependent bioassay indicated that the treated samples enhanced seed germination and seedling growth compared to the untreated wastewater, with the response increasingly aligning with the deionized water control as the dilution progressed. The results demonstrate that photocatalytic treatment significantly reduced the biological toxicity of the wastewater, and the multiparameter bioassay provides a more comprehensive assessment of environmental safety than a single-endpoint test.

### 2.10 Structural Stability and Reusability

The stability and catalytic performance of 40-GCN/CF were tested using 2-NP, 4-NP, and TCP in D.I. water as target pollutants in six cycles (Figure 7). Over the first five cycles, the degradation capacity and photocatalytic activity of 40-GCN/CF remained stable (99% 2-NP, 98.7% 4-NP, and 94.7% TCP in 3 h), indicating a catalyst with a consistent specific surface area and active sites in repeated use. This also suggests the chemical and structural stability of 40-GCN/CF. The degradation activity of 40-GCN/CF decreased slightly in the final cycle (97.2% 2-NP, 94.1% 4-NP, and 92.5% TCP in 3 h).

**Figure 7:**
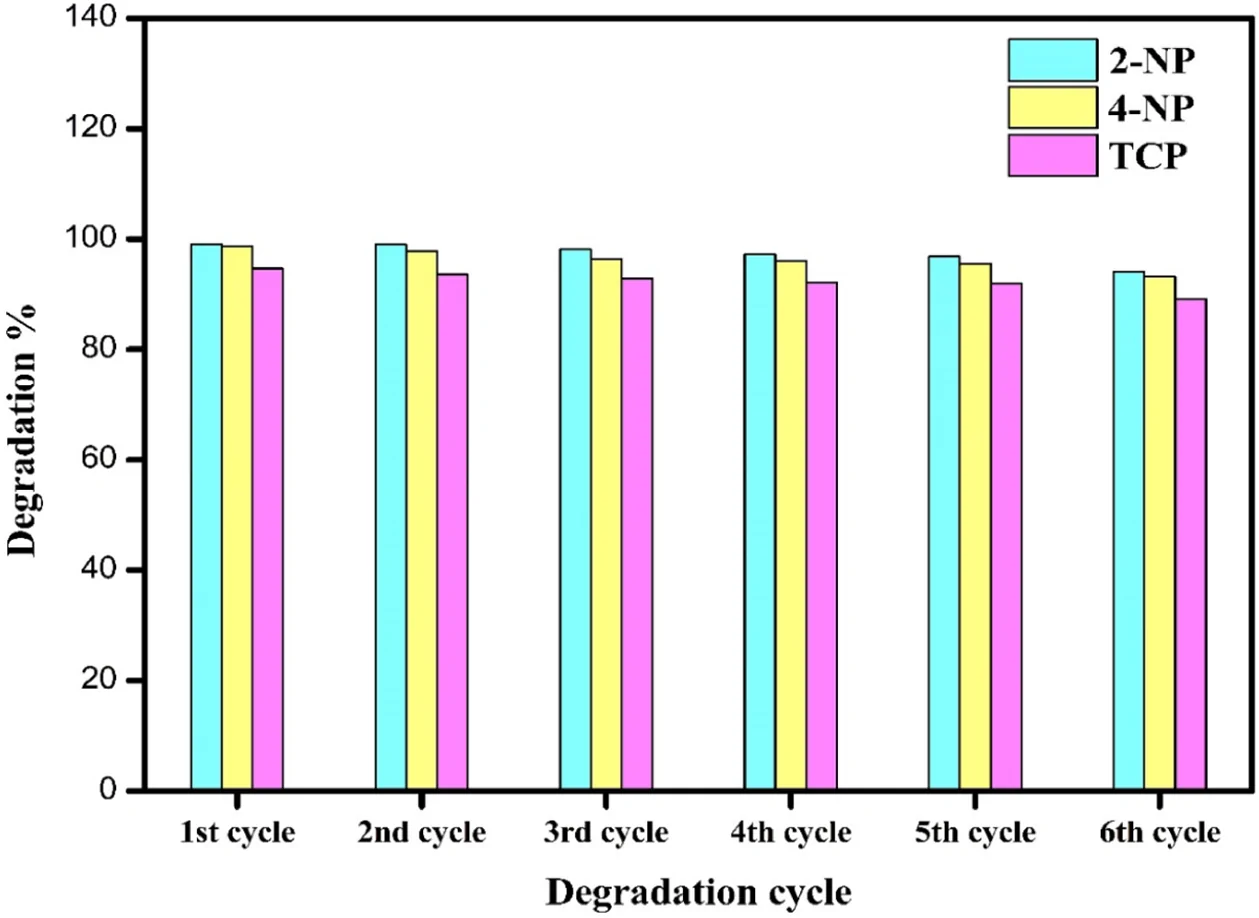
Degradation efficiency test of 40-GCN/CF for six cycles.

The reason behind this may be the irreversible adsorption of phenol on the catalyst, active-site blockage, and, thereby, decreased light absorption. The morphological stability of the prepared photocatalyst was confirmed by FESEM images of 40-GCN/CF after six cycles, which showed no significant difference from the original sample (Figure S9, ESI). XRD measurements before and after degradation were compared. The peak intensity of 40-GCN/CF was constant during photocatalytic degradation (Figure S10, ESI). Similarly, X-ray photoelectron spectroscopy (XPS) survey and high-resolution spectra of the recycled sample indicated the same elemental composition and oxidation states of N, Ca, and F, confirming that the surface chemical environment was retained after cycling (Figure S11, ESI).

### 2.11 Active Radical Species Analysis

Photodegradation of organic pollutants relies on active radical species. The most active free radicals in the redox reaction are O_2_^–^•, OH•, and VB h^+^. Electron spin resonance (ESR) confirmed these active species in the reaction medium (Figure S9 a, b, and c). 5,5-Dimethyl-1-pyrroline N-oxide (DMPO) is widely used to demonstrate free radical involvement in chemical and biological reactions. It detects oxygen-centered radicals like superoxide and hydroxyl. In Figure S12 a (ESI), a 1:1:1:1 intensity characteristic peak confirmed the formation of the DMPO-O_2_^–^• complex and the presence of O_2_^–^• radicals in the reaction medium.^56^ After 5 min of irradiation, low-intense peaks appeared. However, after 20 min, the signals intensified, confirming O_2_^–^• generation in the 40-GCN/CF dispersion. This experimental result agrees with the M-S plot, which shows that the CBM of the GCN molecule is at 1.51 eV, which is more negative than the electrostatic potential needed for O_2_^–^• formation (−0.33 eV vs. NHE).^57^ O_2_^–^• radicals promote degradation and may generate H_2_O_2_, increasing OH• concentration, which is crucial to the reaction mechanism. Figure S12 b (ESI) shows a 1:2:2:1 signal, indicating DMPO–OH• complex formation in the reaction medium. Under prolonged photoillumination, greater exposure times result in increased signal intensity and OH• radical formation. The VBM of CF was 2.3 eV in the M-S plot, which is higher than the electrostatic potential needed for OH• formation (1.99 eV vs. NHE).

The 40-GCN/CF dispersion under visible light showed three 1:1:1 signal peaks after adding TEMPO (2,2,6,6-tetramethylpiperidine-1-oxyl), another radical-trapping agent. This forms a TEMPO-h^+^ complex, which confirms that holes (h^+^) were one of the main active radicals in the water-splitting mechanism and enhanced OH• radical formation.

Adding different active species scavenger molecules to the reaction medium in free-radical scavenging experiments extended the study of the active species. Section 3.4, ESI describes the experimental details. p-BQ, IPA, and TEOA were added to the reaction mixture to trap O_2_^–^•, OH•, and h^+^. The best reaction parameters were kept constant. It was clearly seen in Figure S12 d (ESI) that the presence of p-BQ and IPA reduced the degradation efficiency by more than 50% compared to the normal conditions. This proves O_2_^–^• and OH• are crucial to photocatalysis. TEOA reduced degradation by 20–30%, proving that photoinduced h^+^ was an active species in degradation.

### 2.12 Theoretical DFT Calculations

The geometric structures and electronic properties of pristine GCN and CF layers were examined in the DFT-based theoretical investigation. The GCN and CF layers were structurally optimized first (Figure 8a,b). sp^2^ Hybridized nitrogen (N) atoms are in blue color in (Figure 8a) top view of a GCN sheet. Nitrogen atoms connect graphitic chain carbon atoms (gray color). The cubic unit cell of pure CF atom was optimized (Figure 8b). Calcium atoms connect to negatively charged F atoms (blue colored). The value of lattice parameters obtained from the DFT analysis is 5.413 Å, which is very close to the value derived from the XRD data (5.46 Å).

**Figure 8:**
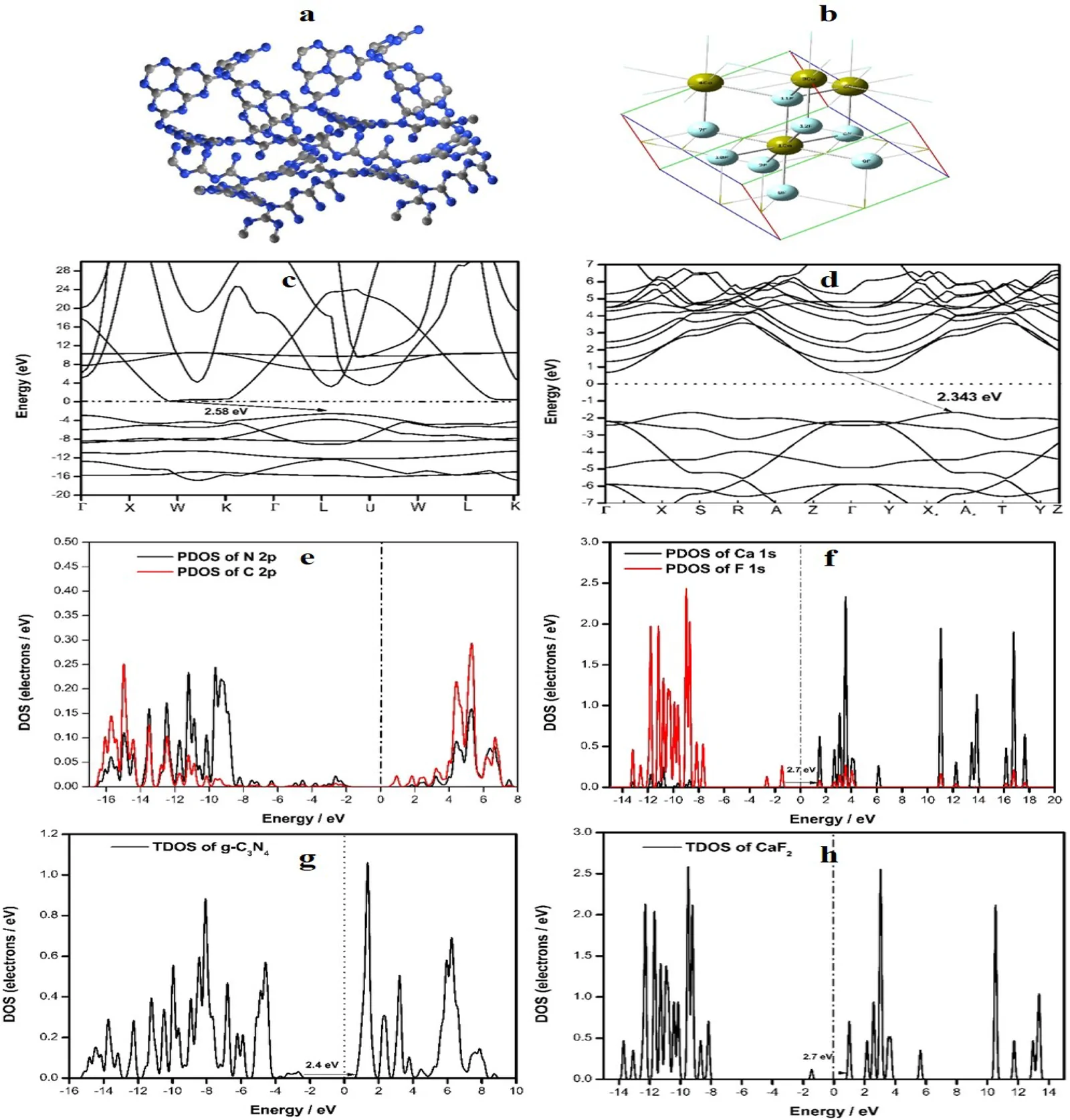
Geometry optimization of a unit crystal: (a) GCN crystal; (b) CF crystal; theoretical calculations: Band structures of pristine (c) GCN and (d) CF; PDOS of (e) GCN and (f) CF; TDOS of (g) GCN and (h) CF.

DFT calculations reveal the electronic energy band structures of the samples. The calculations compared the energy band structures and electronic distributions of pristine GCN and CF. Figure 8c and d show the computed electronic structures. The energy band structures are presented for both pure GCN and CF samples in Figure 8c,d, respectively. The calculated band gap (*E*_g_) values for GCN and CF are observed to be 2.643 and 2.58 eV, respectively. These values align closely with the experimental measurements that were obtained from UV-vis absorbance spectra and Tauc plots. Furthermore, the energy band diagrams reveal that the top valence band (VBT) and the bottom of the conduction band (CBB) for both GCN and CF are positioned at distinct locations, signifying that both samples exhibit characteristics typical of indirect band-gap semiconductors. Unlike direct-gap semiconductors, photoexcited electrons in indirect-gap semiconductors must travel a certain distance in k-space to reach the VB, which reduces carrier recombination and boosts photocatalytic activity.^58^

Figure 8e–h present the partial density of states (PDOS) and total density of states (TDOS) of both pristine GCN and CF nanoparticles. The insight gained from the PDOS calculation (Figure 8e,f) pertains to the charge-transfer mechanism in both nanocatalysts. The VBT of GCN comprised of a combination of s and p orbitals from nitrogen (N) and carbon (C) atoms, while the corresponding bottom of the CB consists of 2p orbitals from nitrogen (N) and carbon (C). Similarly, within the CaF_2_ structure, the Ca^2+^ ion encompasses a vacant 2s orbital in the CBB, while the VBT consists of partially filled 2p orbitals of F atoms. This interplay between the partially filled 2p orbitals of F and the vacant 2s orbitals of Ca facilitates charge transfer between the VBT and CBB.

Moreover, the TDOS (Figure 8g,h) reveals the theoretical band gaps of pure GCN and CF as 2.4 and 2.7 eV, which are in good agreement with the previously determined band gaps from UV–vis spectra and band-structure plots. The GCN/CF heterojunction and N-vacancy-containing heterojunction geometries were developed (Figure S13, ESI), and their total densities of states were analyzed (Figure S14, ESI) to better describe the photocatalyst. The interface models show GCN-CF electronic coupling, while the vacancy-containing heterostructure has a modified electronic distribution that suggests an enhanced interfacial charge redistribution.

### 2.13 Photocatalytic Degradation Mechanism

Based on previous findings, the Type-II and S-schemes are two potential models for interfacial charge separation and migration. Under visible light, GCN and CF can produce photoinduced electrons (e^–^) and holes (h^+^). In the case of coupling GCN with CF, forming a conventional Type-II heterojunction, when exposed to visible light, the photogenerated electrons in the CB of GCN migrate to the CB of CF. Concurrently, the photoinduced holes (h^+^) in the VB of CF are transferred to the VB of GCN. Nevertheless, the accumulated electrons in the CB of CF (−0.169 eV) are insufficient to reduce O_2_ to O_2_^–^• (−0.33 eV versus NHE) since the band potential of the CB is less negative than the standard reduction potential for O_2_/O_2_^–^•. Likewise, VB holes of GCN (1.131 eV) are unable to react with OH^–^ to produce OH• (2.40 eV versus NHE). These observed trends clearly contradict the outcomes of active species trapping experiments and electron spin resonance (ESR) observations. Therefore, a traditional Type-II heterojunction between GCN and CF may not be optimal for the degradation of NPs and TCP. Hence, adopting an S-scheme heterojunction model proves to be a promising approach to elucidate the NP and TCP degradation process.^59,60^ When exposed to visible light, both GCN and CF generate photoinduced electrons and holes. From GCN to CF, an IEF forms. Due to electrostatic attraction between electrons in the CB of CF and holes in the VB of GCN, CB electrons from CF migrate to the VB of GCN at the heterojunction interface. Inside GCN and CF, this transfer minimizes the recombination of electron–hole pairs, enabling the charge carriers to become more spatially separated (Scheme 1a). Concurrently, the charge carriers stored in the CB of GCN and the VB of CF retain their significant reduction and oxidation capabilities, supplying a robust driving force for the prolonged photodegradation reaction.

**Scheme 1:**
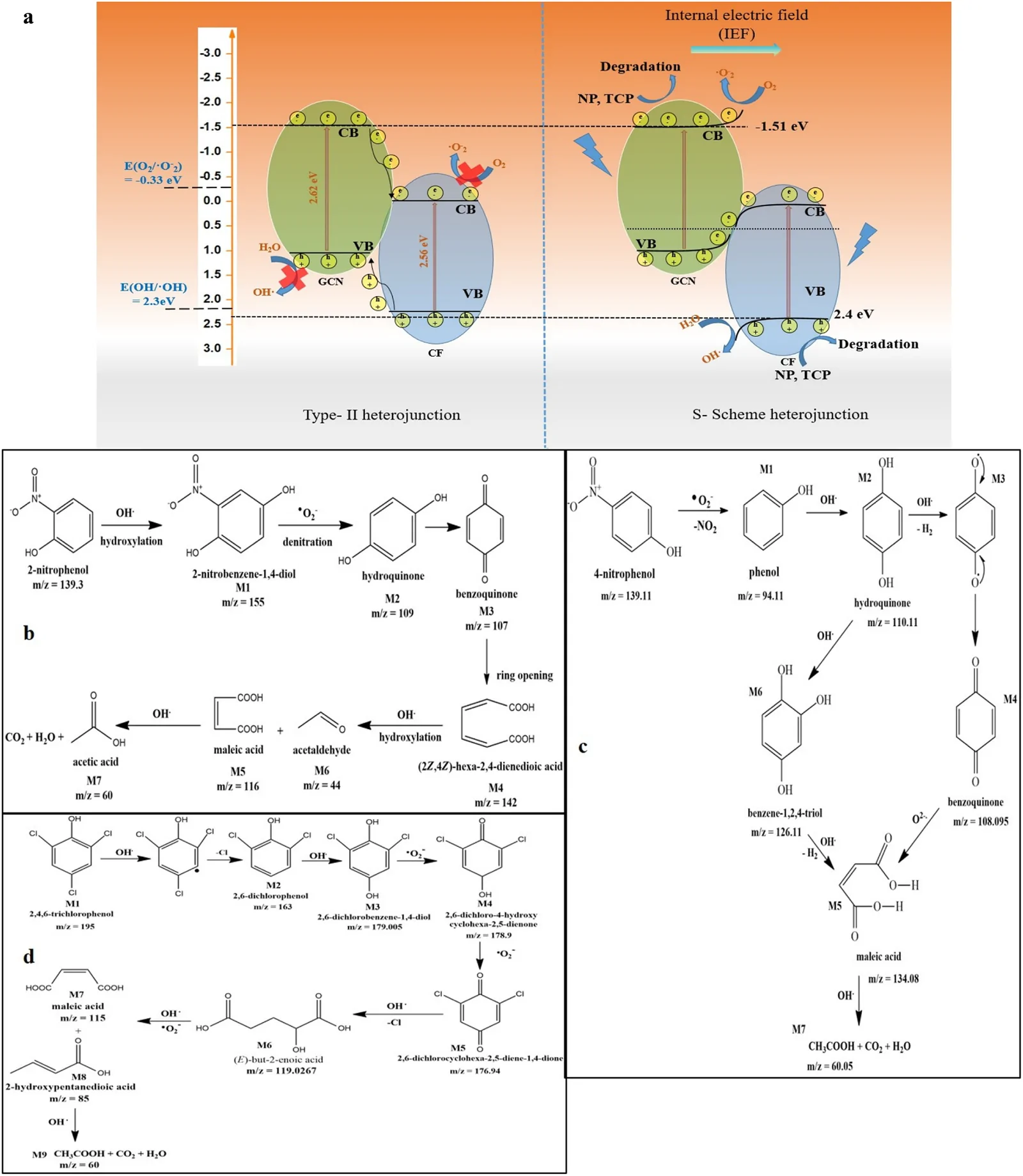
(a) The Possible Mechanisms of Photoexcited Electron–Hole Transport in 40-GCN/CF under Visible Light; (b) Possible Degradation Pathway of 2-NP by 40-GCN/CF; (c) Possible Degradation Pathway of 4-NP by 40-GCN/CF; (d) Possible Degradation Pathway of TCP by

Ultraviolet photoelectron spectroscopy (UPS) was used to study the surface electronic structure of the individual components. The SECO was determined from the UPS analysis (Figure 9) and the work function was calculated from eq 12.

11 $$Work\;function\left(\phi \right)=h\nu -\mathrm{secondary}\;electron\;cutoff\;(\mathrm{SEC}O)\;energy\;\left(E_{cutoff}\right)$$

**Figure 9:**
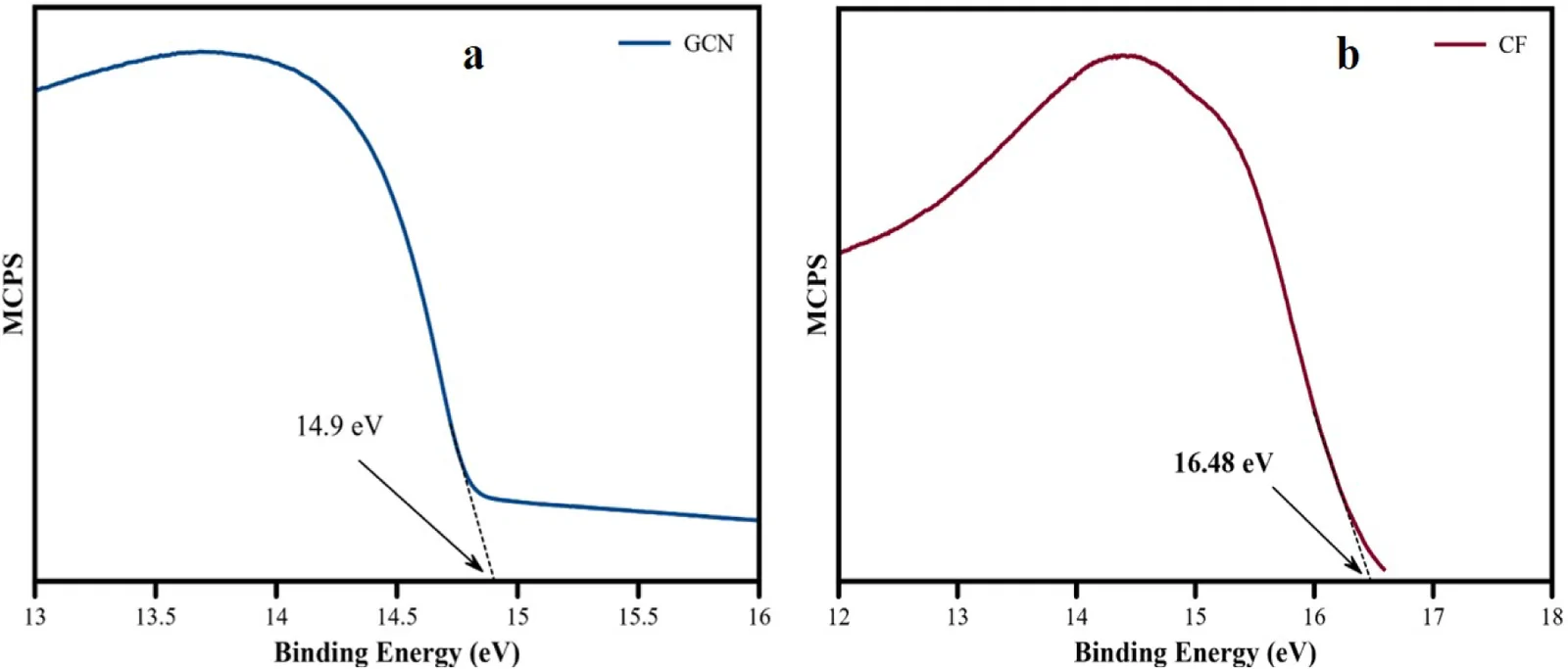
UPS analysis and secondary electron cutoff determination of (a) GCN and (b) CF.

The work function values of GCN and CF were measured to be 6.32 and 4.74 eV, respectively, which indicate a significant difference in the surface Fermi level positions. The contact between the two semiconductors is predicted to lead to electron redistribution from the CF with the lower work function to the GCN with the higher work function until Fermi-level equilibration is reached, inducing interfacial band bending and an internal electric field.^61^ This interfacial charge redistribution further supports the proposed charge-transfer pathway in the GCN/CF heterostructure.

The reduction process uses photogenerated electrons on GCN, while the oxidation process uses photogenerated holes on CF. The h^+^ in the VB of CF can degrade NPs and TCP, supporting active-species capture and ESR results.

12 $$40‐\mathrm{GCN}/\mathrm{CF}+\mathrm{h\nu}\to 40‐\mathrm{GCN}/\mathrm{CF}\left({e_{\mathrm{CB}}}^{-}+{h_{\mathrm{VB}}}^{+}\right)$$

13 $$\mathrm{GCN}\left({e_{\mathrm{CB}}}^{-}\right)+O_{2}\to {O_{2}}^{-}•$$

14 $$\mathrm{CF}\left({h_{\mathrm{VB}}}^{+}\right)+H_{2}O\to \mathrm{OH•}+H^{+}$$

15 $${O_{2}}^{-}•+\mathrm{Pollutant}\;\left(\mathrm{NPs}\;\mathrm{and}\;\mathrm{TCP}\right)\to \mathrm{Degraded}\;\mathrm{products}$$

16 OH•+Pollutants(NPsandTCP)→Degradedproducts

Structural, optical, and electrochemical evidence was correlated to study the photocatalytic electron-transfer pathway. Mott–Schottky analysis indicated that GCN is an n-type semiconductor and CF is a p-type semiconductor, and that the composite exhibits heterojunction behavior consistent with interfacial band bending. The reduced PL intensity, increased photocurrent, and decreased EIS resistance of GCN/CF imply more efficient separation and transport of the photogenerated charge carriers.

Direct ultrafast verification through in situ irradiated XPS or femtosecond transient absorption spectroscopy was not carried out in this work. However, this is still an important direction for further research. Even though direct in situ charge-tracking measurements were not carried out, the evidence that was gathered from band alignment, Mott–Schottky analysis, photocurrent response, PL quenching, TRPL prolongation, and reactive-species trapping collectively lend support to the S-scheme charge-transfer pathway that was proposed.

### 2.14 Photodegradation Pathways

To elucidate the degradation process of NPs and TCP, LC-MS/MS analysis was conducted, leading to the establishment of a plausible pathway, as depicted in Scheme 1b, c, and d. Detailed information on the respective m/z peaks and individual MS spectra can be found in ESI, Tables S7, S8, and S9. The comprehensive formation pathway of intermediate products during the degradation of 2-NP on 40-GCN/CF is depicted in Scheme 2b. The electron-donating nature of the hydroxyl group of the benzene ring promotes the electrophilic attack of OH• in the ortho and para positions, ultimately resulting in the formation of 3-nitrobenzene-1,4-diol (Figures S15, S16, S17, ESI). Given the strong electrophilic nature of the nitro group, it is exceptionally challenging to remove it from the benzene ring. The formation of hydroquinone (M2) serves as evidence of the potent oxidizing ability of OH• induced by 40-GCN/CF. The generated nitroso radical can react with photoexcited electrons to produce nitrite ions, which are subsequently oxidized to nitrate ions by O_2_^–^• in the solution. In the next step, hydroquinone (M2) reacts with the superoxide radical, leading to the production of benzoquinone (M3). Furthermore, the interaction of intermediates with OH• radical promotes ring opening and transformation into oxygenated aliphatic compounds, including maleic acid (M5), acetaldehyde (M6), and acetic acid (M7). This process ultimately leads to the generation of final products such as CO_2_ and H_2_O.

**Scheme 2:**
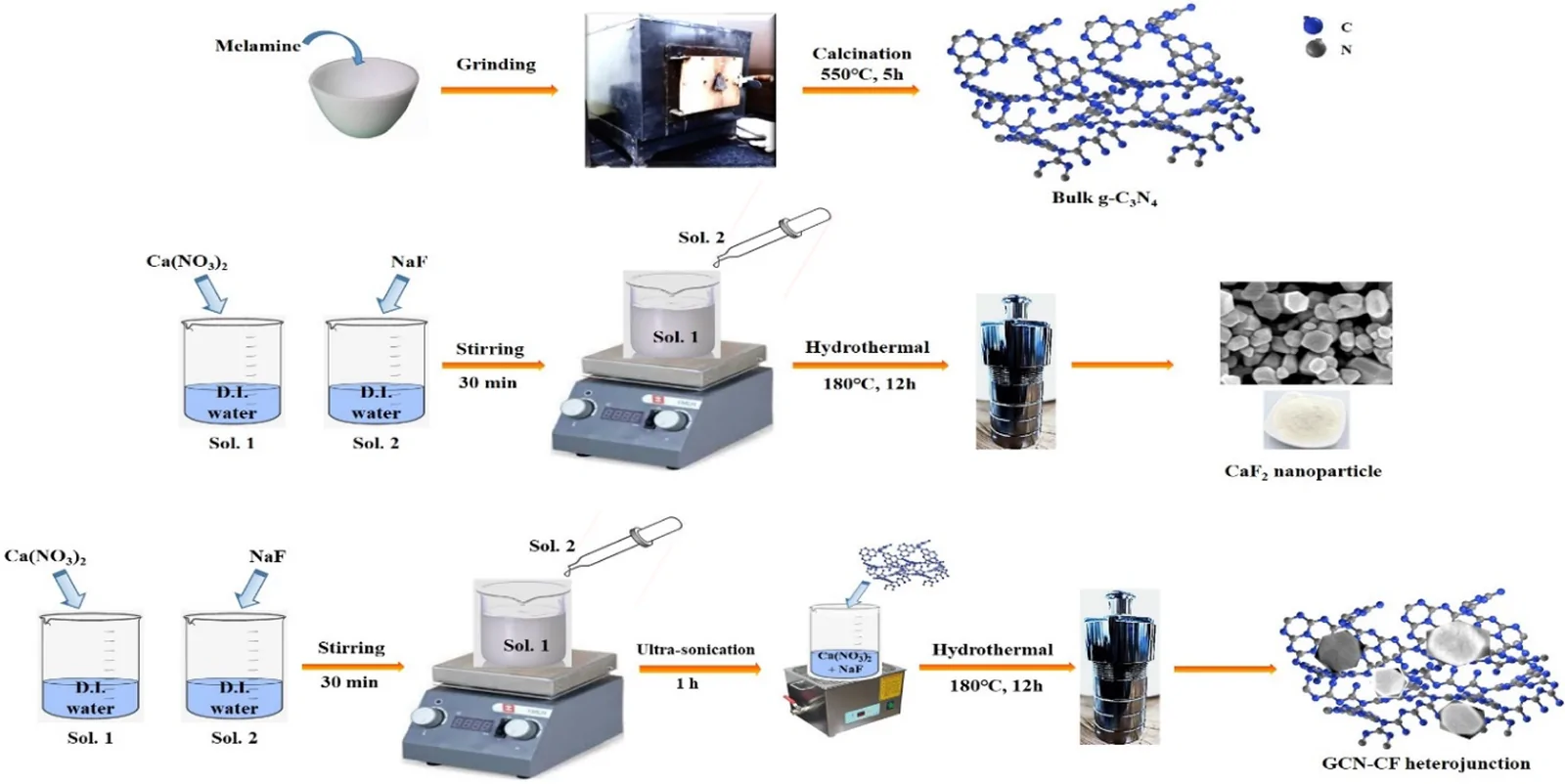
Synthesis Procedure of Pure GCN, CF, and GCN/CF Heterojunction

Throughout the degradation of 4-NP, five primary and intermediate products were identified (Figures S18, S19, S20, ESI), exhibiting peaks at m/z values of 134.08, 126.11, 110.11, 94.11, and 60 (Scheme 2c). It is important to emphasize that the m/z value of 139.11 represents the molecular ion peak of 4-NP (M1). Due to the electron-donating nature of the hydroxyl group on 4-NP, it functions as an ortho–para positional substituent, while the nitro group on the benzene ring, owing to its electron-withdrawing property, acts as a meta-positioning group.^62^ When 4-NP is exposed to free radicals, it undergoes a denitration reaction, resulting in the formation of phenol (M1) through free-radical oxidation. Phenol is subsequently degraded to produce p-benzoquinone (M4) and maleic acid (M5). Conversely, 4-NP can also undergo a transformation into benzene-1,2,4-triol (M6, m/z = 126) before being subsequently reduced to maleic acid. The benzene ring of p-benzoquinone was cleaved and oxidized to form maleic acid (M5, m/z = 116). The presence of acetic acid (M7) was also detected in the reaction mixture, which might have been produced during further oxidation of maleic acid by active radical species.

Table S8 of ESI shows the intermediate products generated during the TCP degradation of 2,4,6-TCP as detected by LC-MS (Figures S21, S22, ESI). Highly reactive OH• and O_2_^–^• are generated, attack the ortho and para positions of TCP, and convert to an unstable anion. Afterward, dechlorination takes place and produces M2 (m/z = 163) (Scheme 2d). The OH• radicals again oxidize M2 and convert it into M3 (m/z = 179.005) due to hydroxylation. The photoexcited electrons from the CB of GCN take part in electron oxidation and M3 is converted to M4 (m/z = 178.9). A further series of oxidations takes place, which produces M5 (m/z = 176.94). The OH• and O_2_^–^• initiate the ring-opening reaction, and subsequently dechlorination forms the straight-chain product M6 (m/z = 119.0267). Further degradation produces smaller organics like maleic acid (M7, m/z = 115) and acetic acid (M9, m/z = 60). Final degradation mineralizes these into CO_2_ and H_2_O. Table S10 (ESI) shows the degradation efficiency of previously reported g-C_3_N_4_ photocatalysts for pollutants.

## 3 Conclusion

This study developed and analyzed g-C_3_N_4_ sheets with different CaF_2_ weight percentages, named 10-GCN/CF, 20-GCN/CF, 30-GCN/CF, 40-GCN/CF, and 50-GCN/CF nanocomposites. This simple synthesis process used calcination and solvothermal methods for catalyst development and to mineralize 2-NP, 4-NP, and TCP in aqueous systems using an advanced oxidation process (AOP) following photocatalysis. In comparison to either standalone GCN or CF, the synthesized GCN/CF composite demonstrates significantly improved degradation of aromatic-substituted phenol compounds through photocatalysis under visible-light conditions. GCN/CF nanocomposites were characterized using XRD, FT-IR, FE-SEM, TEM, and EDX, confirming sheet-like GCN and cubic CF crystals in different semiconductor nanocomposites. Compared to pure GCN and CF, the 40-GCN/CF composite showed excellent photocatalytic activity, performing 99% degradation for 2-NP, 98.7% for 4-NP, and 94.7% for TCP. The 40-GCN/CF composite degraded somewhat higher than 39% of phenolic pollutants in polluted and turbid Hooghly River water, according to degradation rates. The ideal weight percentage of CF, band gap energy, surface area, and light absorption of GCN/CF improve charge carrier separation. This efficient electron–hole pair separation increases ROS (reactive oxygen species) production, photodegradation, and photocurrent generation. Photocatalytic degradation pathways for aromatic phenols were proposed. This study synthesizes and characterizes GCN/CF for the photocatalytic degradation of various nitrophenolic pollutants, paving the way for its use in photocatalytic environmental pollution remediation.

## 4 Materials and Methods

### 4.1 Catalyst Synthesis

#### 4.1.1 Construction of g-C_3_N_4_ Nanosheets

A simple polymerization technique of melamine powder was carried out to construct graphitic carbon nitride (g-C_3_N_4_) nanosheets. In this process, around 5 g of pure melamine powder was taken in an alumina boat and kept in a muffle furnace under a lid cover. The polymerization was done at 550 °C with a 5 °C/min ramp rate for four hours. After completion of the polymerization, a pale yellowish powder was obtained. This yellow sample was collected and labeled as bulk GCN.

#### 4.1.2 Fabrication of CaF_2_ Nanoparticles

3.97 mmol of Ca­(NO_3_)_2_ was dissolved in 20 ml of D.I. water. This solution was labeled as Sol 1. Another aqueous solution was made by dissolving 11.3 mmol of NaF in 20 ml of D.I. water and marked as Sol 2. Sol 2 was added dropwise to Sol 1 under vigorous magnetic stirring. After mixing, the stirring was continued for another 30 min until a milky whitecolored solution appeared. At this stage, the solution was kept in a Teflon-lined hydrothermal reactor and placed in a muffle furnace at 180 °C for 12 h. After completion of the reaction, the reactor was gradually cooled to reach room temperature. The solid CaF_2_ powder was separated and washed rigorously four to five times with D.I. water and ethanol and dried in an oven at 65 °C for 10 h. The pure CaF_2_ was labeled as CF.

#### 4.1.3 Synthesis of GCN/CF Heterojunction

0.5 g of GCN powder was added to 30 ml of D.I. water and sonicated for 1 h for homogeneous dispersion. On the other hand, different weight % of the Ca-precursor salt (10%, 20%, 30%, 40%, and 50% of the weight of g-C_3_N_4_) and the required F-precursor (NaF) were added to D.I. water to prepare CaF_2_ with varying Ca content by the same procedure as stated earlier for CaF_2_ synthesis. After rigorous washing with ethanol and D.I. water, the GCN/CF samples were labeled according to the wt % of CaF_2_ added (10-GCN/CF, 20-GCN/CF, 30-GCN/CF, 40-GCN/CF, and 50-GCN/CF). The detailed synthesis processes of bulk GCN, CF, and GCN-CF are described in Scheme 2.

## Supplementary Material



## Data Availability

Data will be made available on request.

## Notes

Additional details on catalyst characterization and photocatalytic experiments, including BET–BJH analysis, FTIR spectra, elemental mapping, photoelectrochemical measurements, UV–Vis spectra, LC–MS/MS data and degradation intermediates, TOC analysis, phytotoxicity results, radical-trapping experiments, post-reaction FE-SEM and XRD characterization, Mott–Schottky plots, and supplementary tables and calculations (PDF)
