# Supplementary material for: Synergistic N‑Vacancy and S‑Scheme Engineered CaF2/g‑C3N4 Heterojunction for Photocatalytic Aromatic Phenol Degradation: DFT, Phytotoxicity, and Environmental Impact
Source: ACS Omega. 2026 Sep 9;11(37):56031–48. doi: 10.1021/acsomega.6c06038 (PMC13613868; doi:10.1021/acsomega.6c06038)
Supplement: Supplementary file 1 [file ao6c06038_si_001.pdf]

Electronic Supplementary Information (ESI)

Synergistic N-Vacancy and S-Scheme Engineered CaF_2/g-C_3N_4 Heterojunction for Photocatalytic Phenol-derivatives degradation: DFT, Phytotoxicity, Environmental Impact

Dwaipayan Dhar [a], Sonali Sengupta [a] *

[a] Department of Chemical Engineering, Indian Institute of Technology Kharagpur, Kharagpur 721302, India

* Corresponding author.

Email address: sonalis.iitkgp@gmail.com (Sonali Sengupta)

sonalis@che.iitkgp.ac.in

ORCID ID: 0000-0002-2748-1372

S 1.1 Chemical reagents:

Melamine [$C_3H_6N_6$, 98%], 2-nitrophenol, 4-nitrophenol, 2, 4, 6-trichlorophenol were purchased from Loba Cheme. 2-propanol (CH_3CH (OH) CH_3, 99%), sodium fluoride [NaF, 98%] and calcium nitrate hydrate [Ca $(NO_3)_2$, $4H_2O$], benzoquinone (BQ) [99%], isopropanol (IPA) [99%], ethylene diammine tetra-acetic acid disodium (EDTA-2Na) [98%] and ethanol [98%] were purchased from Merck.

S 2.1 Characterization techniques:

The structural morphology and the nanostructure of the synthesized catalysts and heterostructures were analyzed by a field emission scanning electron microscope (FESEM) of FESEM-MERLIN and transmission electron microscope (TEM) facility of JEM-2100 TEM (JEOL, Japan). The Fourier transform-infrared spectroscopy (FT-IR) instrument of the PerkinElmer Spectrum 100 model was used to observe the presence of different chemical functional groups and their changes in the catalyst samples. The crystallinity and phase purity were determined by X-ray powder diffraction (XRD) analysis of various catalysts and composites by a PAN analytical diffractometer (model: PW-3050/60, UK), using Cu-Kα radiation at 40 kV and 30 mA. The pore size determination and BET surface area distribution of different catalyst samples were calculated by N_2 adsorption-desorption isotherm with Quantrachrome AUTOSORB1 (UK) make BET surface area measurement instrument. A ULVACPHI 5000 versaprobe II instrument with an Al Kα electrode was used to carry out X-ray photoelectron spectroscopy (XPS) to observe the different chemical states of individual chemical constituents of catalyst samples. UV photoelectron spectroscopy (UPS) (PHI 5000 Versaprobe III, Japan) was used to analyze the work function of the catalysts. The optical characteristics of individual catalyst samples were obtained by UV-Vis spectrum analysis by a

UV-Vis spectrophotometer of Analytical Technology, India. The recombination time of charge carrier and luminescence properties were characterized by a Hamamatsu R928 photomultiplier detector with He–Cd laser as the excitation source operating at 325 nm with an output power of 50 mW.

S 3.1 Preparation of simulated wastewater and photocatalytic activity test:

The photocatalytic degradation reactions of 2-nitrophenol (2-NP), 4-nitrophenol (4-NP) and 2, 4, 6- tri nitrophenol (TCP) were carried out in a compact glass reactor equipped with the visible light source. Typically, different amounts of catalyst samples (0.1 to 0.6 g of GCN, CF, and GCN/CF samples) were mixed with 15 ppm aqueous solutions of 2-NP, 4-NP, and TCP. The mixtures were ultrasonicated for 1h in dark conditions to reach the adsorption-desorption isotherm. After that, the solution was transferred to a glass reactor inside, with an annular space where a 100W xenon lamp with a 400nm cutoff filter was installed inside a sealed tube. The light irradiation was carried out for up to 3 h with a continuous air supply from the bottom of the reactor in order to well-agitate the reactant mixture, and air acts as one of the reactants for degradation. During the reaction, the periodical withdrawal of 5 ml of the liquid sample from the reactor was done to record the concentration variation of pollutants. The withdrawn sample was centrifuged to separate the catalyst particles from it. After separation, the concentration changes of NPs and TCP at different time intervals were quantified in a UV-Vis spectrophotometer.

The influence of pH in the medium was explored by adding 0.1M NaOH solution to the mixture. The effect of catalyst dosing and the impact of other water matrices on the degradation efficiency of catalyst samples were studied by varying the initial catalyst loading (0.1g to 0.6g) and changing the type of water for the reaction mixture (replacing D.I water with tap water of our laboratory and polluted Hoogly river water).

The photostability of the catalyst samples was tested after 6 cycles of degradation. The experiments were conducted under the same conditions mentioned earlier. The separated catalyst samples, after centrifugation, were washed with D.I. water and ethanol. After drying, the regenerated catalysts were re-dispersed in the nitrophenol aqueous solution and photoreaction was carried out.

S3.2 Toxicity Estimation:

The phytotoxicity tests were conducted in petri dishes lined with filter paper, a straightforward and economical method. Mung bean (*Vigna radiata*) seeds were chosen for this toxicity evaluation. The experiments included control (distilled water only), different wastewater sample (Di water, tap water and Hoogly river water mixed with phenols) and treated wastewater samples, with 4 mL of each solution applied to the filter paper in 90 mm petri dishes. Each dish contained twenty seeds, sealed with parafilm to prevent evaporation. The seeds were incubated at room temperature, with three replicates for each treatment. After a 5-day incubation period, the germinated seeds were collected, and the root length and wet weight were measured to assess the wastewater toxicity. The seed germination rate (%) was determined by the equation S1:

$$\text{Seed germination rate \%} = \frac{\text{number of germinated seeds}}{\text{total number of seeds}} \times 100\ \% \quad \text{.... (S1)}$$

The germination index (%) was determined by comparing the seed germination and root elongation in the sample to the control using the following equation S2:

$$\text{The germination index (\%)} = \frac{G_S \times L_S}{G_C \times L_C} \quad \text{............(S2)}$$

where G_S and L_S are seed germination rate (%) and root length (cm) for the 40-GCN/CF treated wastewater samplea. G_C and L_C represent the values for the corresponding controls receiving photocatalytic treatment.

S 3.3 Electrochemical measurements:

Different electrochemical measurements, like photocurrent density and electrochemical impedance spectra (EIS) were carried out in an electrochemical workstation (CHI7089, CH Instruments, USA). A fluorine-doped tin oxide (FTO) plate was used as a working electrode or anode. 10 mg of different catalyst samples (GCN, CF and GCN/CF) were mixed with 10 ml of absolute ethanol. 5μL of Nafion solution was added to the solution mixture and sonicated for 30 min to get a well-dispersed mixture. The solution was drop casted on the conductive side of the FTO plate and dried at 50 °C for 2 h. The platinum electrode and Ag/AgCl electrode were used as cathode and reference electrode, respectively. A 1M of KOH solution was used as the working electrolyte. All the potential data obtained from the workstation were calibrated by using the formula [Zhang et al., 2020]

$$E_{RHE} = \left(E_{\frac{Ag}{AgCl}} + 0.098V\right) + 0.059pH \qquad \text{(S3)}$$

For the present measurements, the electrolyte pH was 6.0, and the Ag/AgCl reference electrode potential was taken as 0.19 V vs NHE. Therefore, the pH correction term was:

$$0.059 \times 6.0 = 0.354\ \text{V} \qquad \text{(S4)}$$

Thus, the total conversion factor from Ag/AgCl to NHE at pH 6.0 was:

$$0.19 + 0.354 = 0.544\ \text{V} \quad \text{(S5)}$$

The flat-band potential of GCN measured versus Ag/AgCl was:

$$E_{fb,Ag/AgCl}\text{=[insert measured value] V}$$

The corresponding potential versus NHE was calculated as:

$$E_{fb,NHE}\text{=[insert measured value]+0.544} \quad \text{(S6)}$$

$$E_{fb,NHE}\text{=[final value] V vs NHE}$$

S 3.4 Scavenger experiment:

There are three major active species that are responsible for photocatalytic degradation. Superoxide (O_2^-), hydroxyl (OH•) and holes (h^+) are the three radicals that are generated during the light irradiation on the catalyst reactant mixture. Isopropyl alcohol (IPA), benzoquinone (BQ) and EDTA-2Na are the scavengers for OH•, O_2^- and h^+ radicals, respectively. Under the same reaction condition that was mentioned earlier, each of the scavenger reagents is mixed with the reaction mixture and catalyst at 1mmol amount and the reaction is carried out. The degradation percentage was recorded separately for each scavenger test to observe the effect of the scavenger and to detect the most effective active species.

S 3.5 Computational methodology:

The crystal structure and electronic configuration of pure GCN and CF nanoparticles were developed using DFT simulation and calculation on the Quantum Espresso package. The calculation was executed by using the Generalized Gradient Approximation (GGA) functional Perdew–Burke–Ernzerhof (PBE) as the exchange-correlation function. The relaxed unit cell of GCN and CF molecule and the different coordinates of the atoms were calculated on a Monkhorst-Pack k-point grid using the Broyden–Fletcher–Goldfarb–Shanno (BFGS) algorithm with an optimization threshold of 0.001 Ry/ Bohr. The SCF (self-consistent field) calculation was carried out on a 2 × 2 × 1 Monkhorst–Pack *k*-point grid. After geometry optimization and

SCF convergence calculation, the density of state (DOS) for both the GCN and CF were established to understand the electronic distribution inside the atomic structure.

S 3.6 LC-MS analysis specification:

The reaction intermediates of 2-NP, 4-NP, and TCP degradation were identified by liquid chromatography-mass spectroscopy analysis. The LC–MS system (Waters, USA) coupled to a Micromass Quattro Micro triple-quadruple mass spectrometer (Micromass, Manchester, UK) with MassLynx software. The degradation sample was injected into the ES ionization source using a syringe pump at a flow rate of 10 μl/min. For the analysis of the intermediate, the instrument was operating in the negative ion mode with a capillary voltage of 3 kV and cone voltage of 30 V. An electrospray source block temperature of 90 °C and desolvation temperature of 250 °C was used. The data were acquired in the MS scanning mode with the scan range of 10–1800 (*m*/z), the scan time was 0.5 s and the scan delay time was 0.1 s.

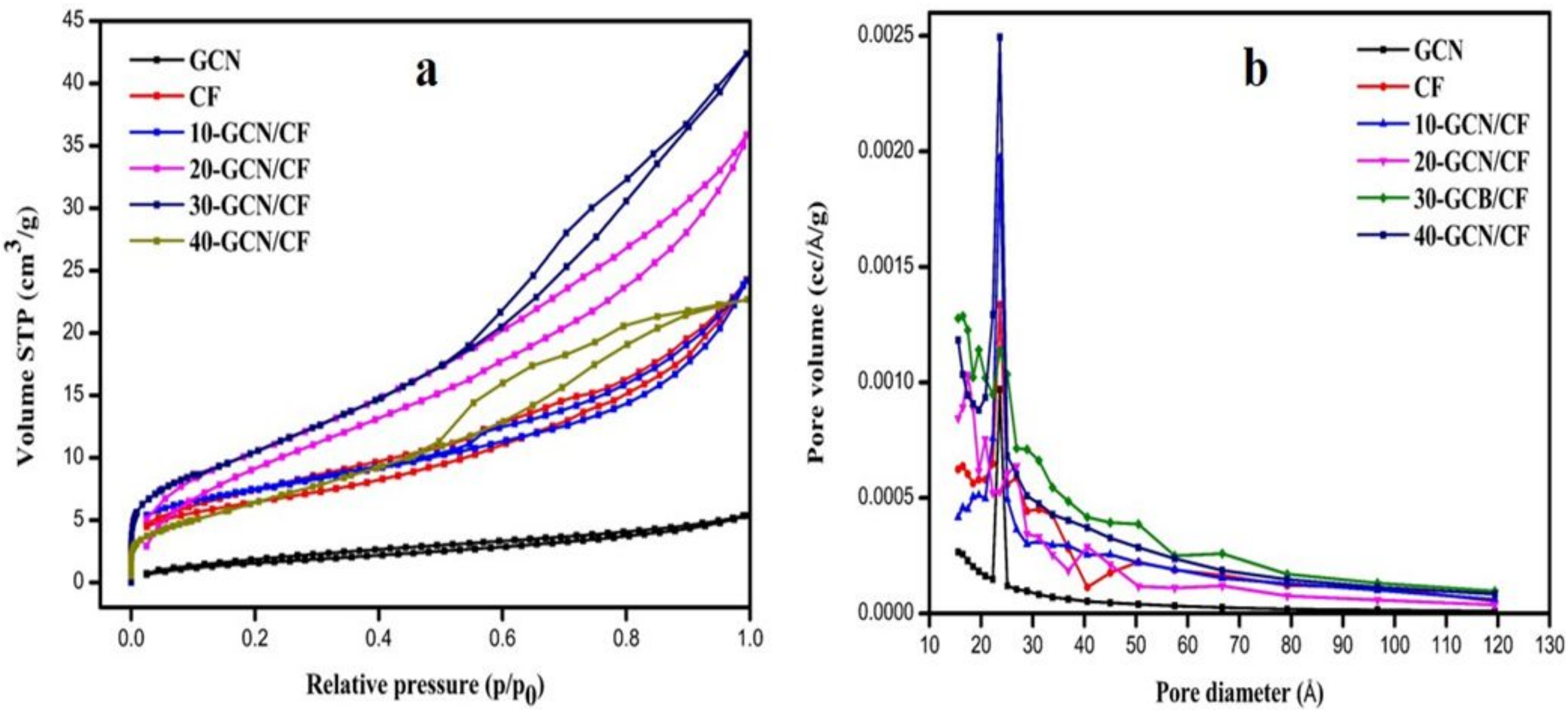

Fig. S1: BET analysis results, (a) N_2 adsorption-desorption isotherm and (b) BJH pore size distribution of pure GCN, CF and GCN/CF heterojunctions

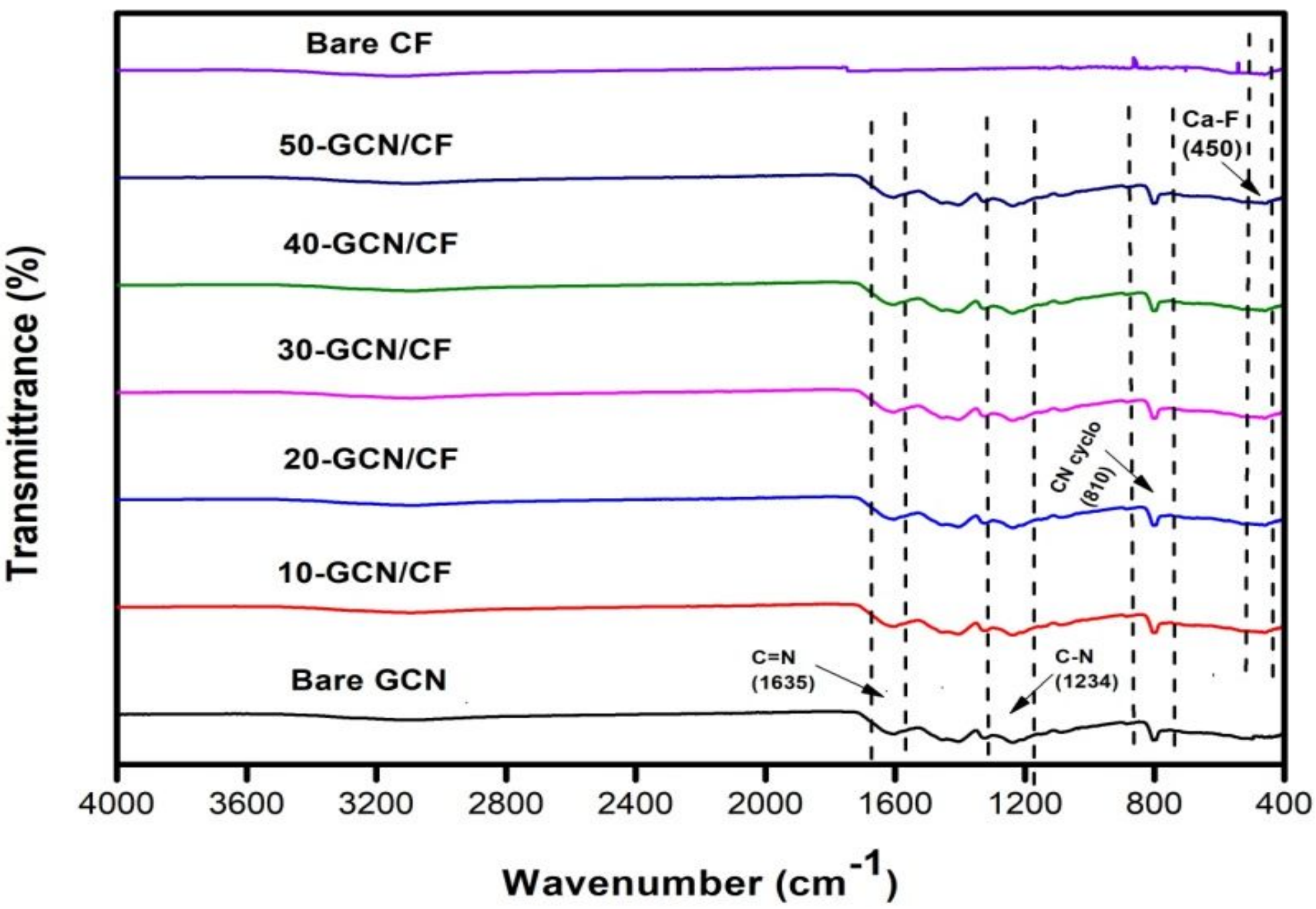

Fig. S2: FTIR spectrum of pure GCN, CF and all GCN/CF heterojunctions

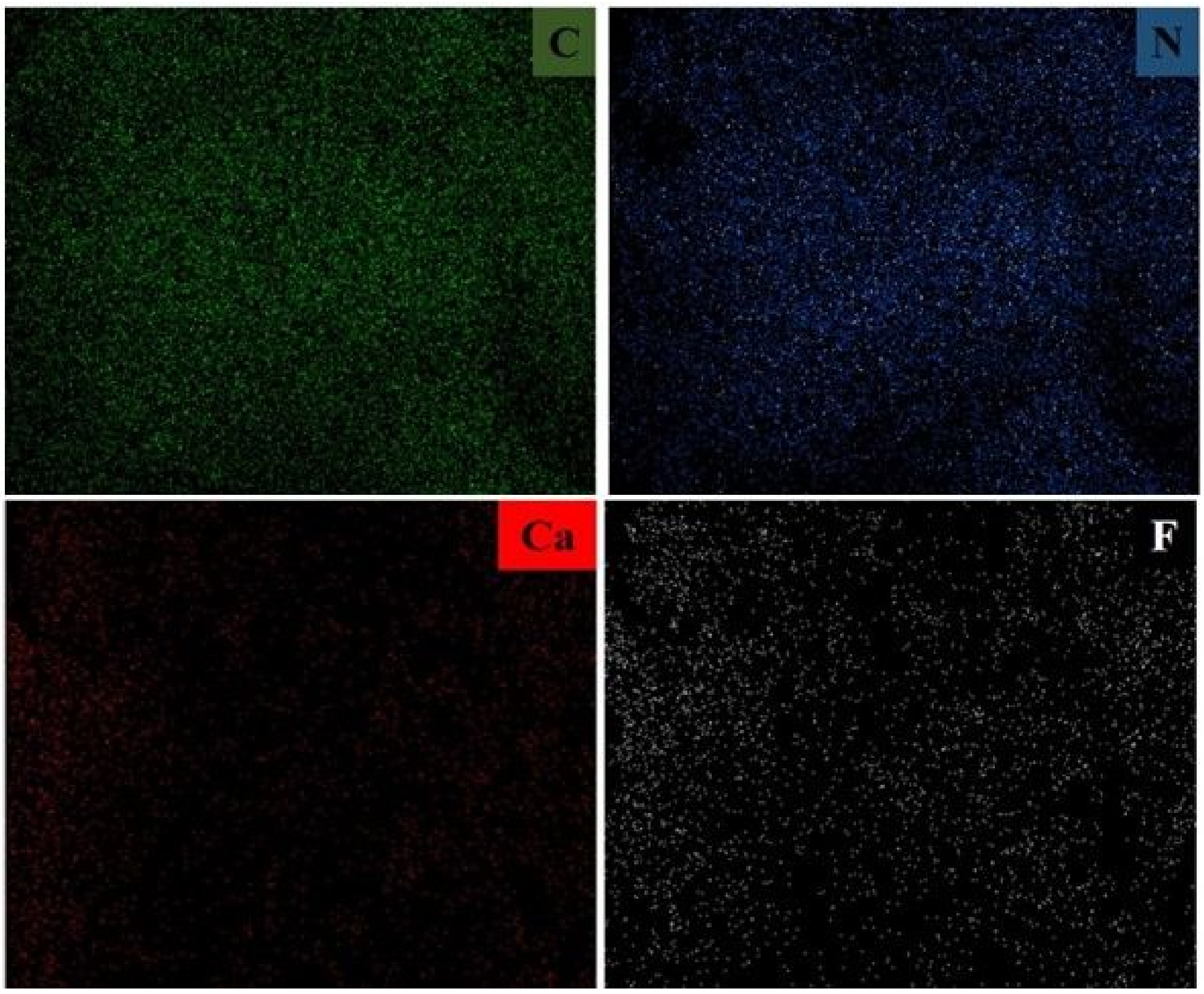

Fig. S3: EDX elemental distribution of 40-GCN/CF

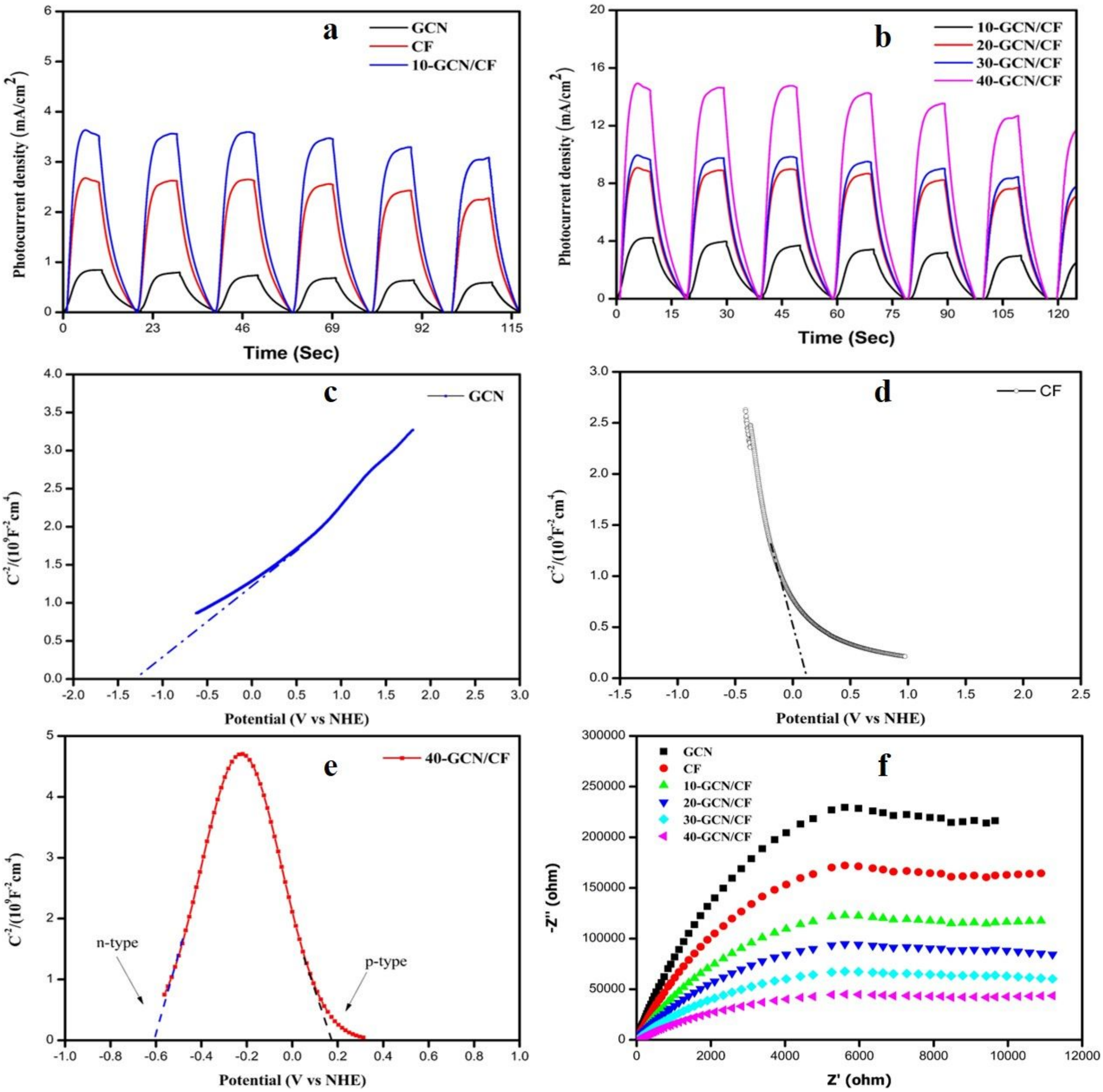

Fig. S4: (a, b) Photocurrent response density of pure GCN, CF and all the GCN/CF heterojunctions; Mott-Schottky plots of (c) n-type GCN, (d) p-type CF, (e) 40-GCN/CF (p-n heterojunction); (f) EIS Nyquist plot of as-synthesized samples

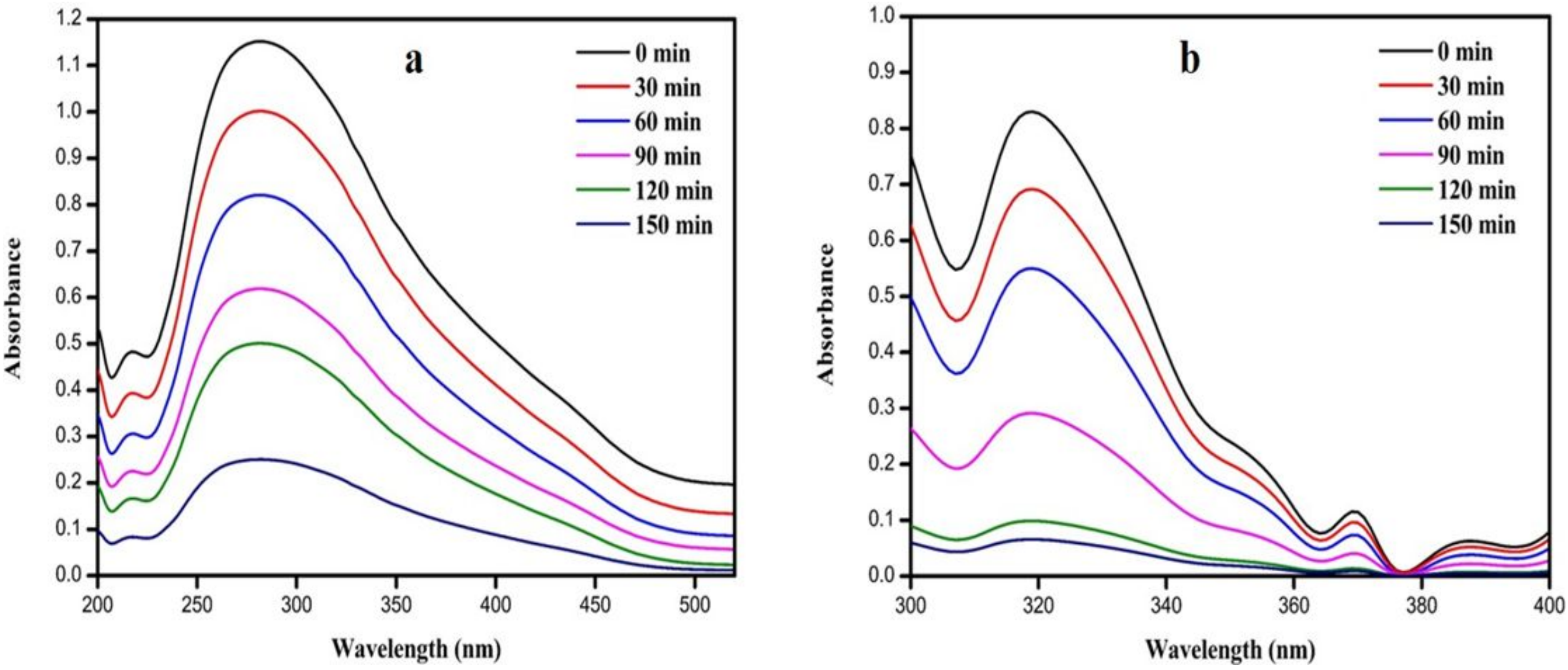

Fig. S5: Time-dependent UV-VIS spectra of (a) 2-NP and (b) TCP solution over 40-GCN/CF catalyst [2-NP and TCP concentration = 15 ppm, catalyst loading = 50 mg/L and 58 mg/L for 2-NP and TCP respectively]

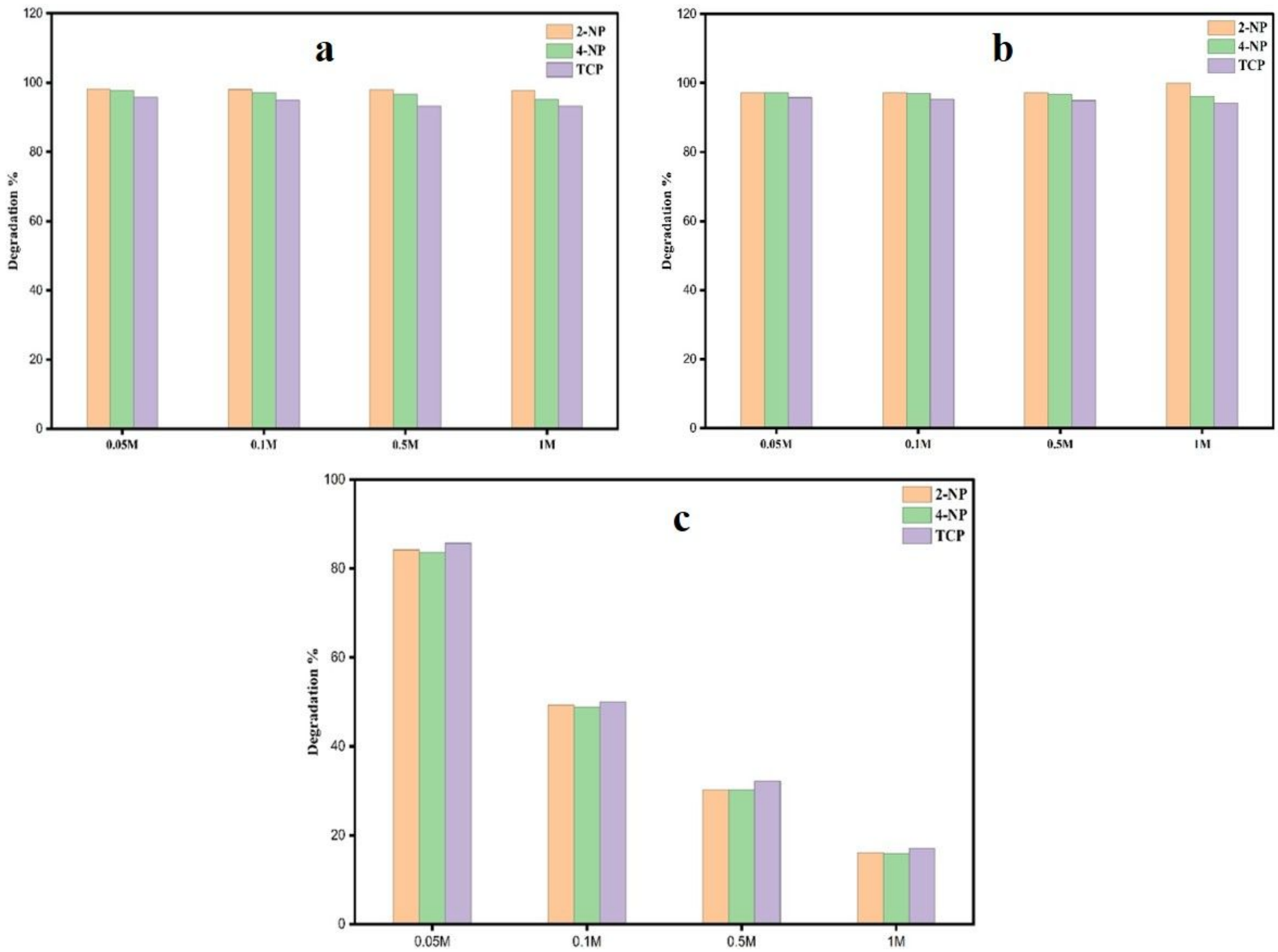

Fig S6: common ion effect in the range of 0.05 M to 1 M concentration of (a) SO_4^{2-}, (b) Cl^- and (c) PO_4^{3-}

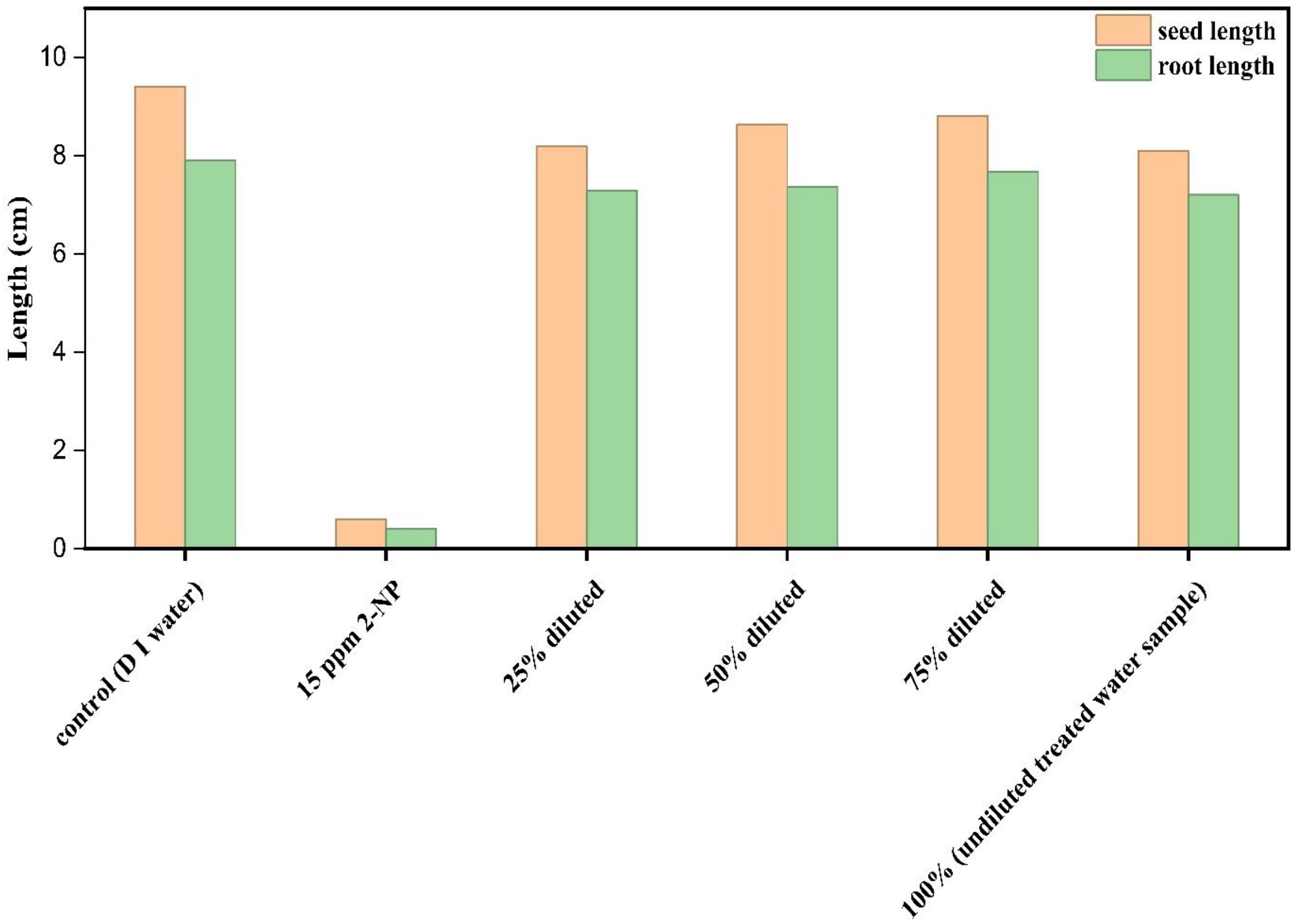

Fig S7: Seed (yellow) and root (green) length of mung bean after 5-days exposure of Wastewater sample with different dilution

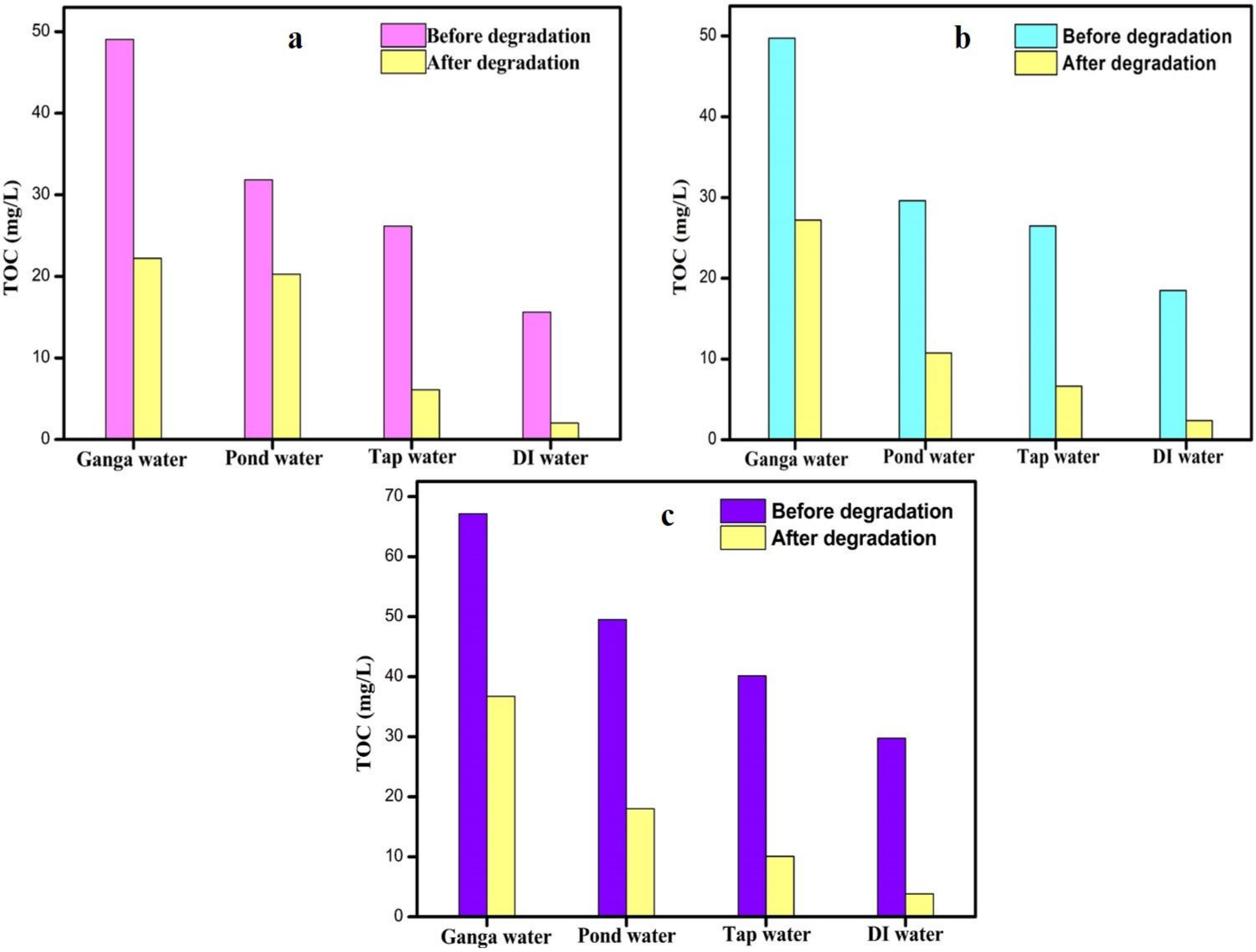

Fig. S8: Total organic carbon removal efficiency of 40-GCN/CF in different wastewater matrix for (a)2-NP, (b) 4-NP and (c) TCP

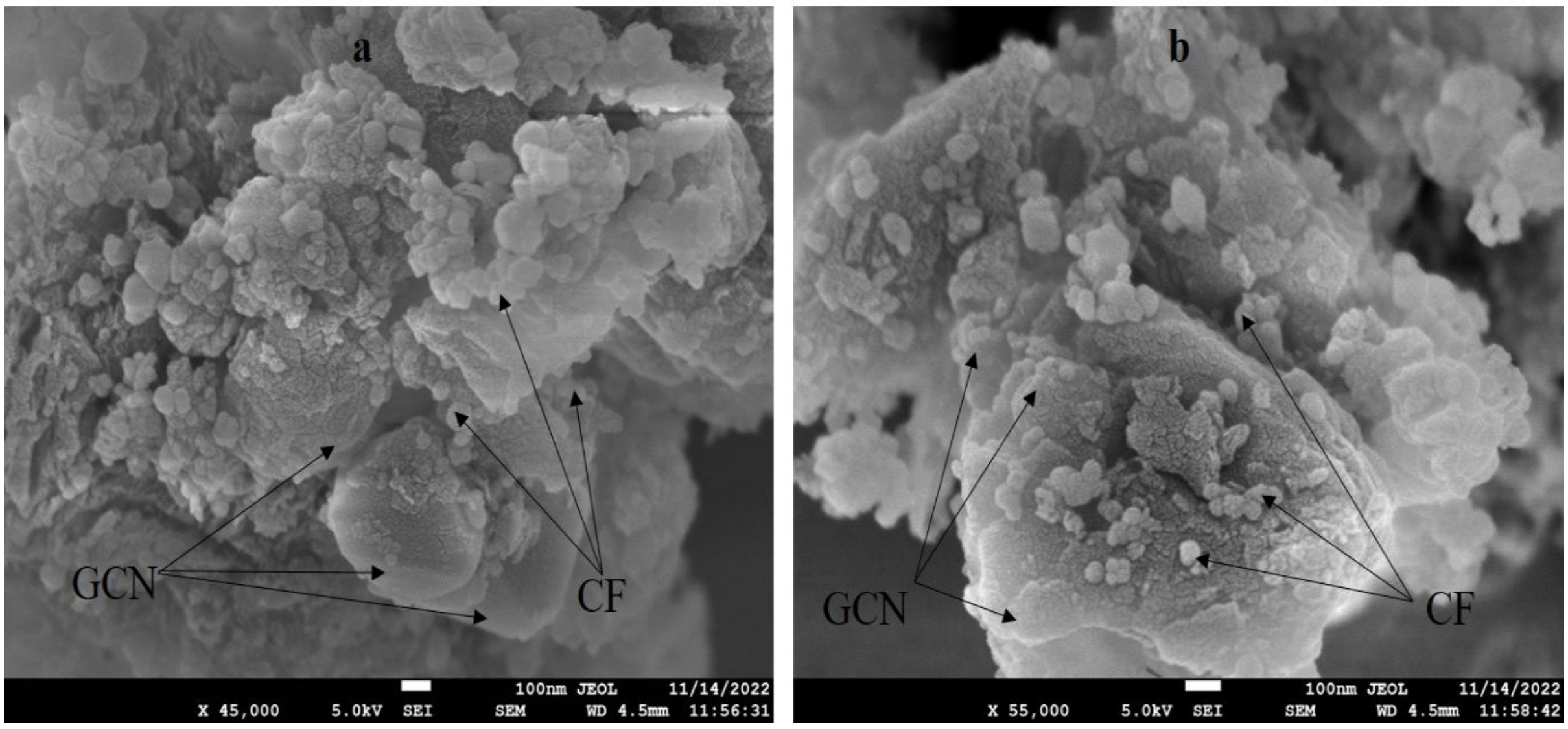

Fig. S9: (a,b) FESEM images of 40-GCN/CF after 6 degradation cycles

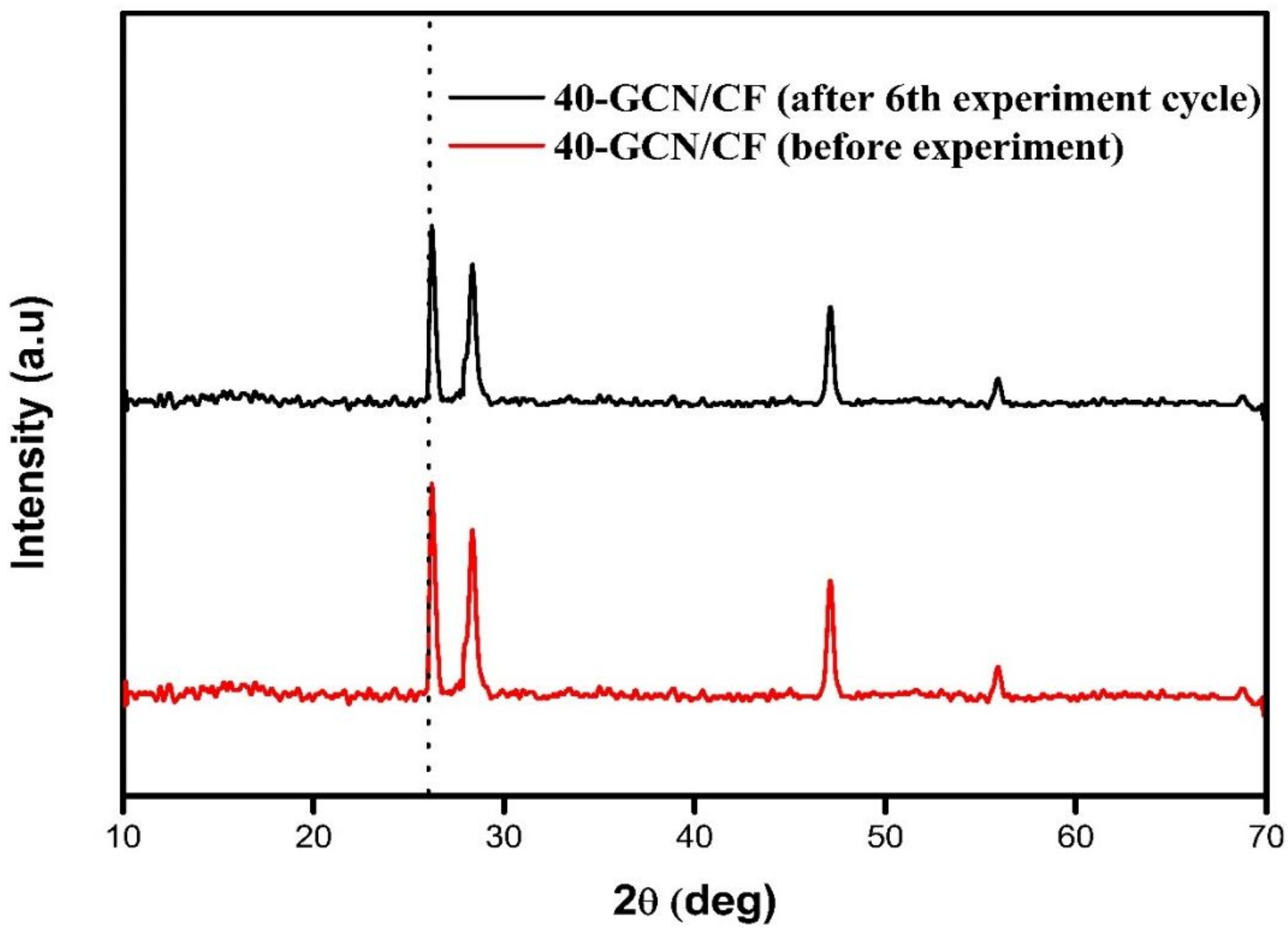

Fig. S10: comparison of XRD analysis of 40-GCN/CF before and after 6 degradation cycles

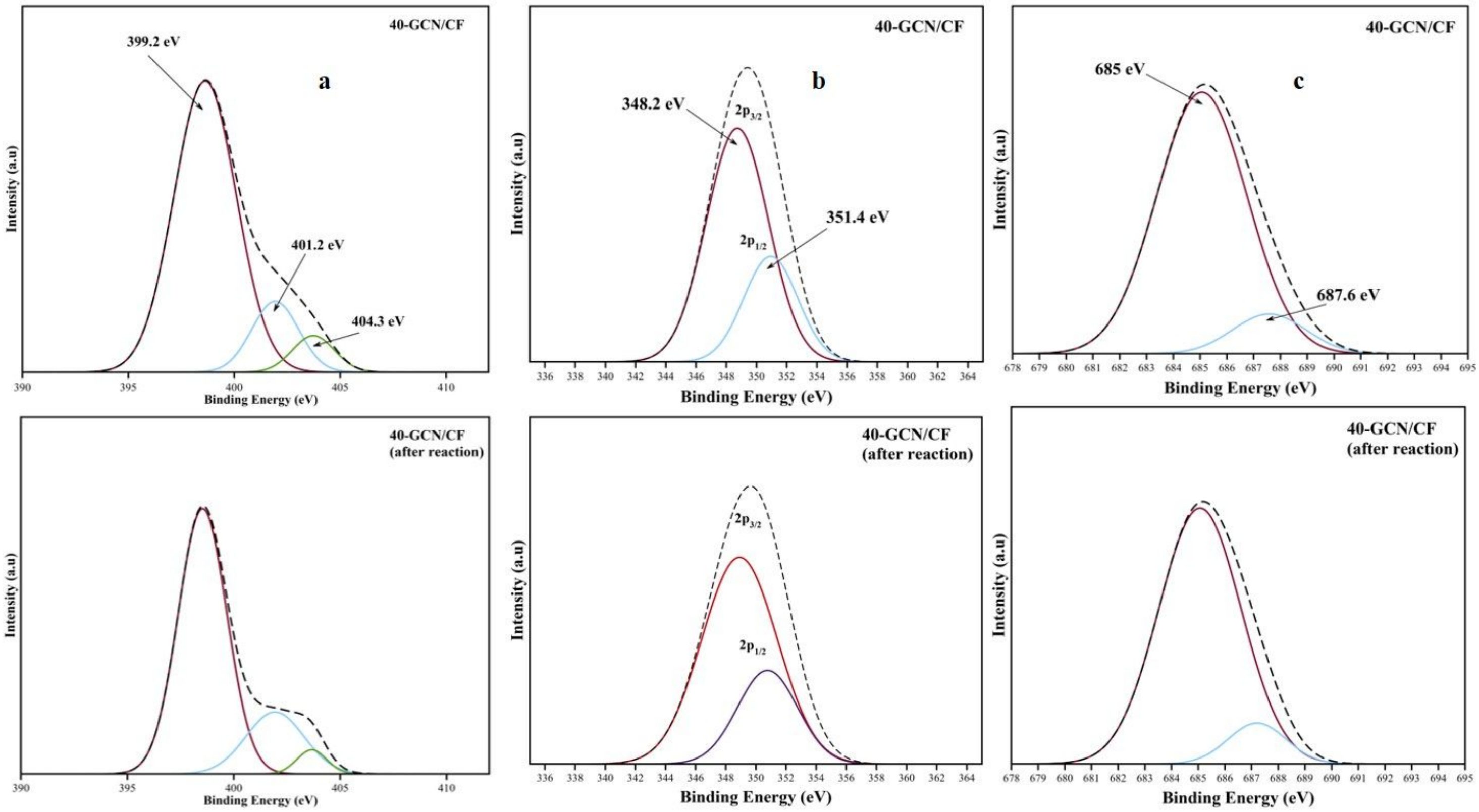

Fig. S11: comparison of XPSanalysis of 40-GCN/CF before and after 6 degradation cycles,

(a) N 1s, (b) Ca 2p and (c)F 1s

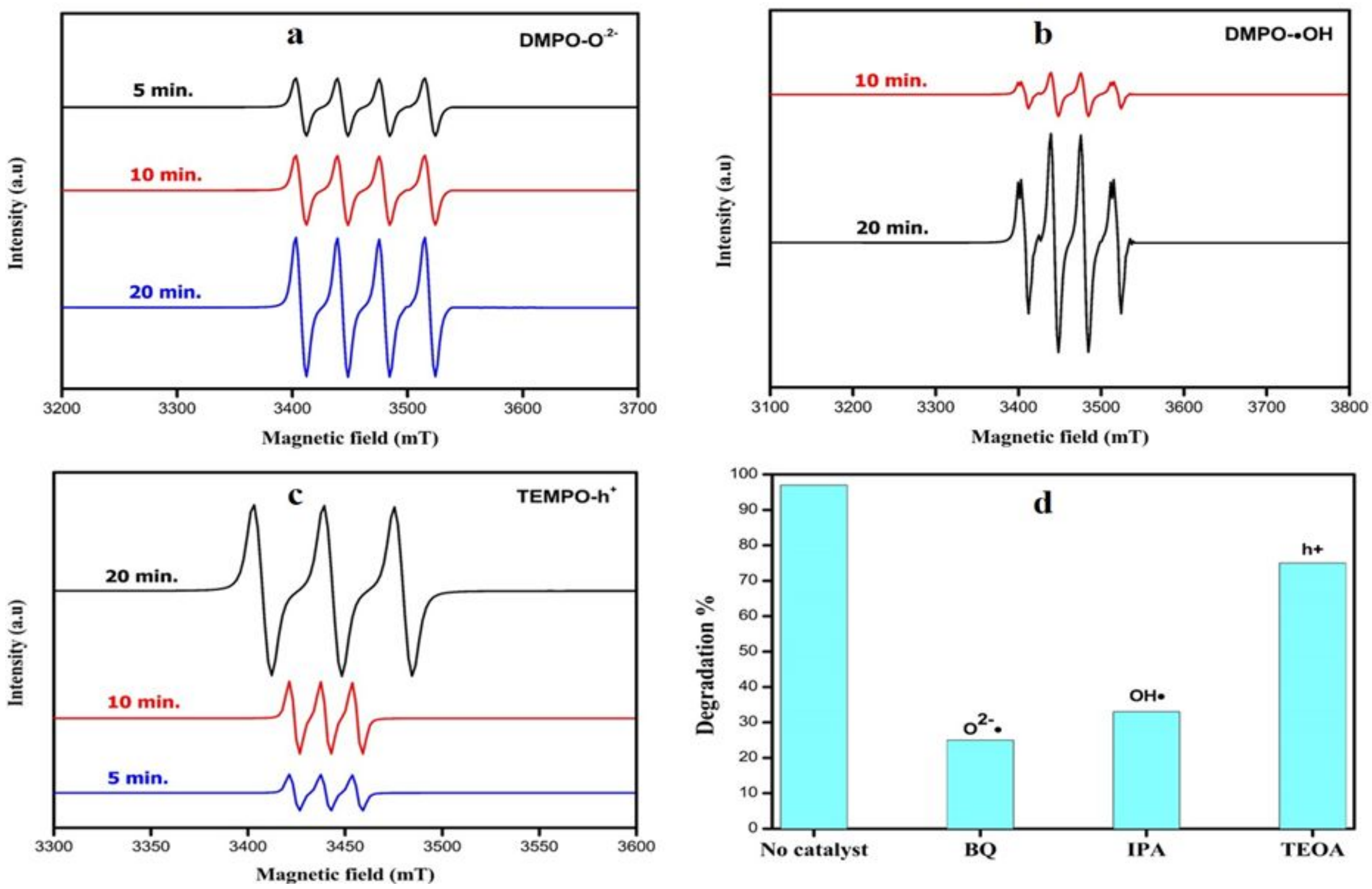

Fig. S12: electron spin resonance (ESR) spectra of radical trapped by different trapping agents in 40-GCN/CF aqueous dispersion under visible light irradiation: (a) DMPO-O_2^{-}• formed, (b) DMPO-OH• formed, (c)TEMPO-h+ formed [40-GCN/CF = 20 mg/L, DMPO = 50mM, TEMPO = 50mM, light irradiation time = 10 min], (d) effect of various scavengers on photocatalytic degradation

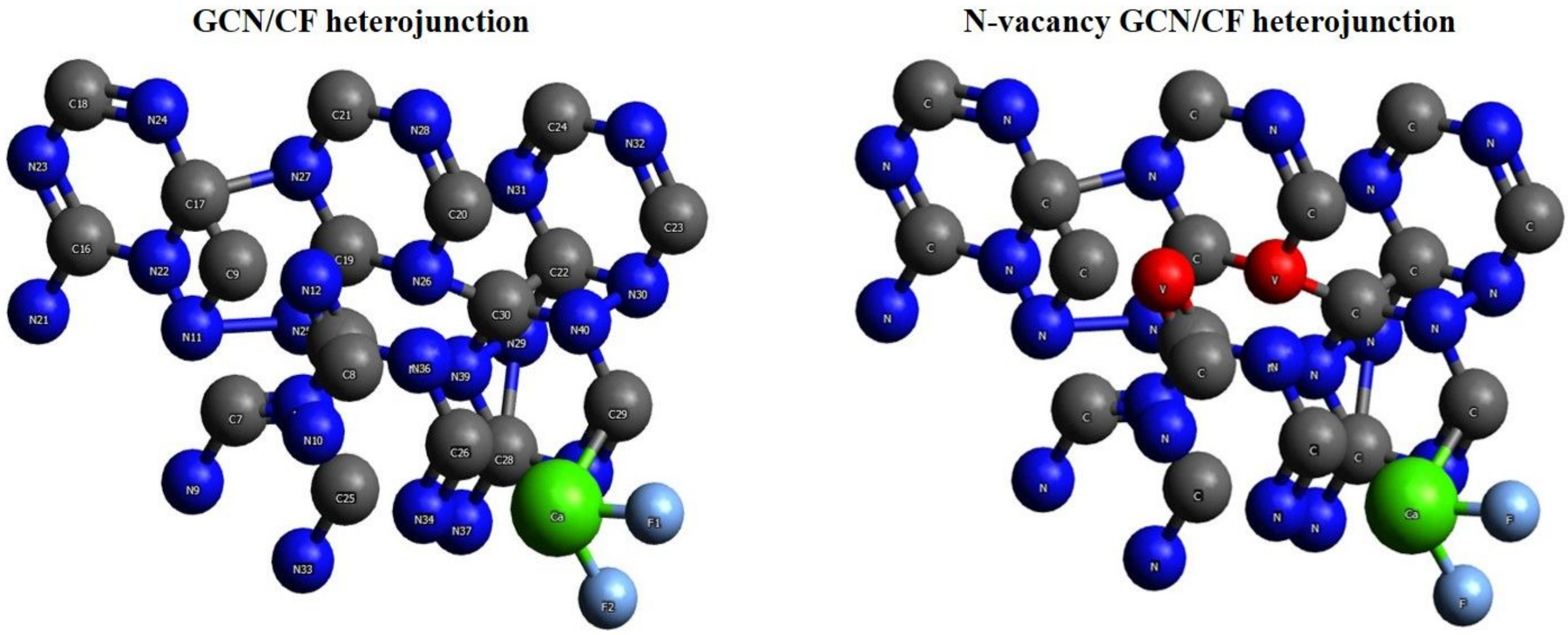

Fig S13: optimized geometric structure of GCN/CF and N-vacancy (red spots) containing GCN/CF

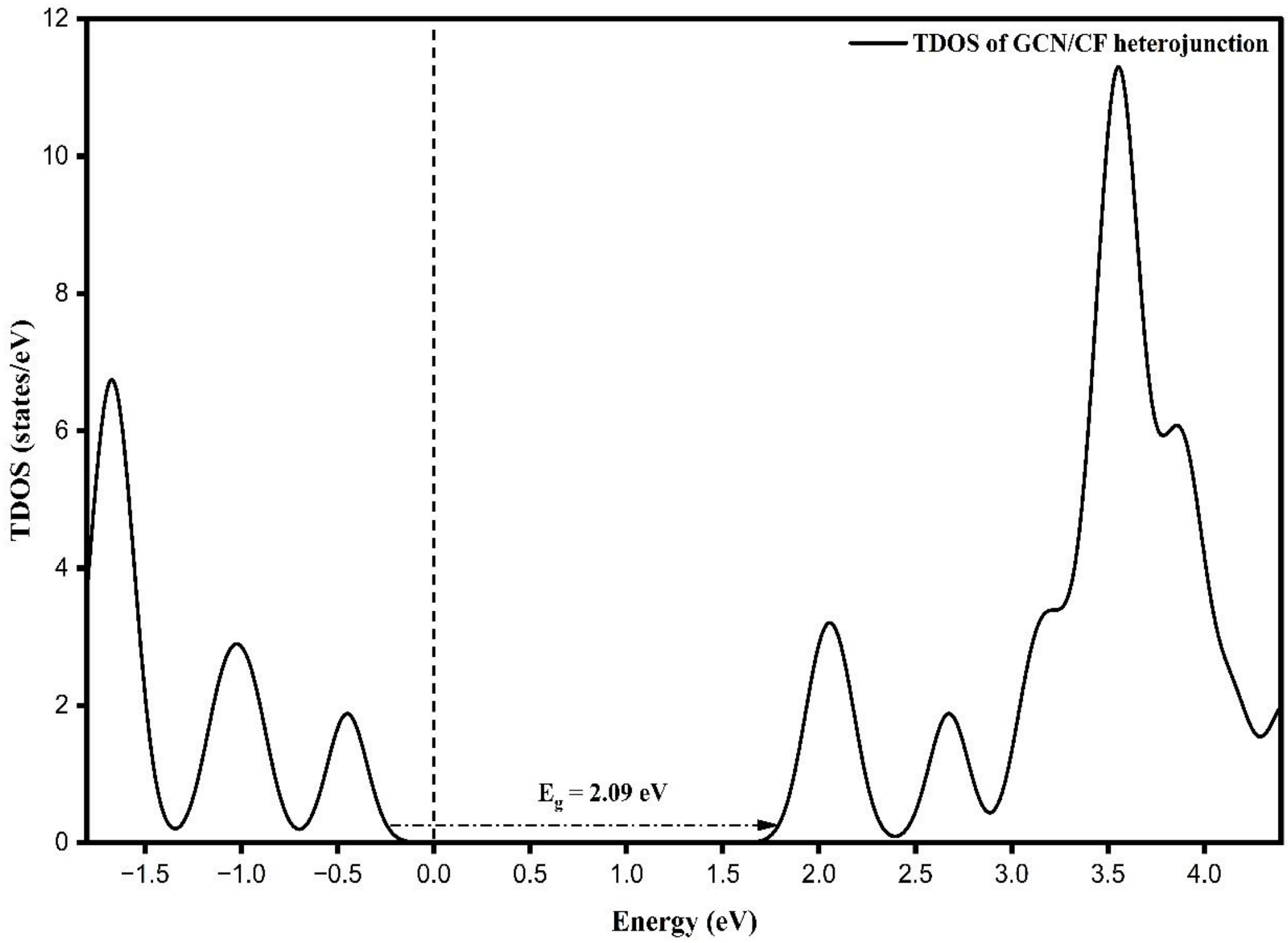

Fig S14: Total density of state (TDOS) of N-vacancy containing GCN/CF heterojunction

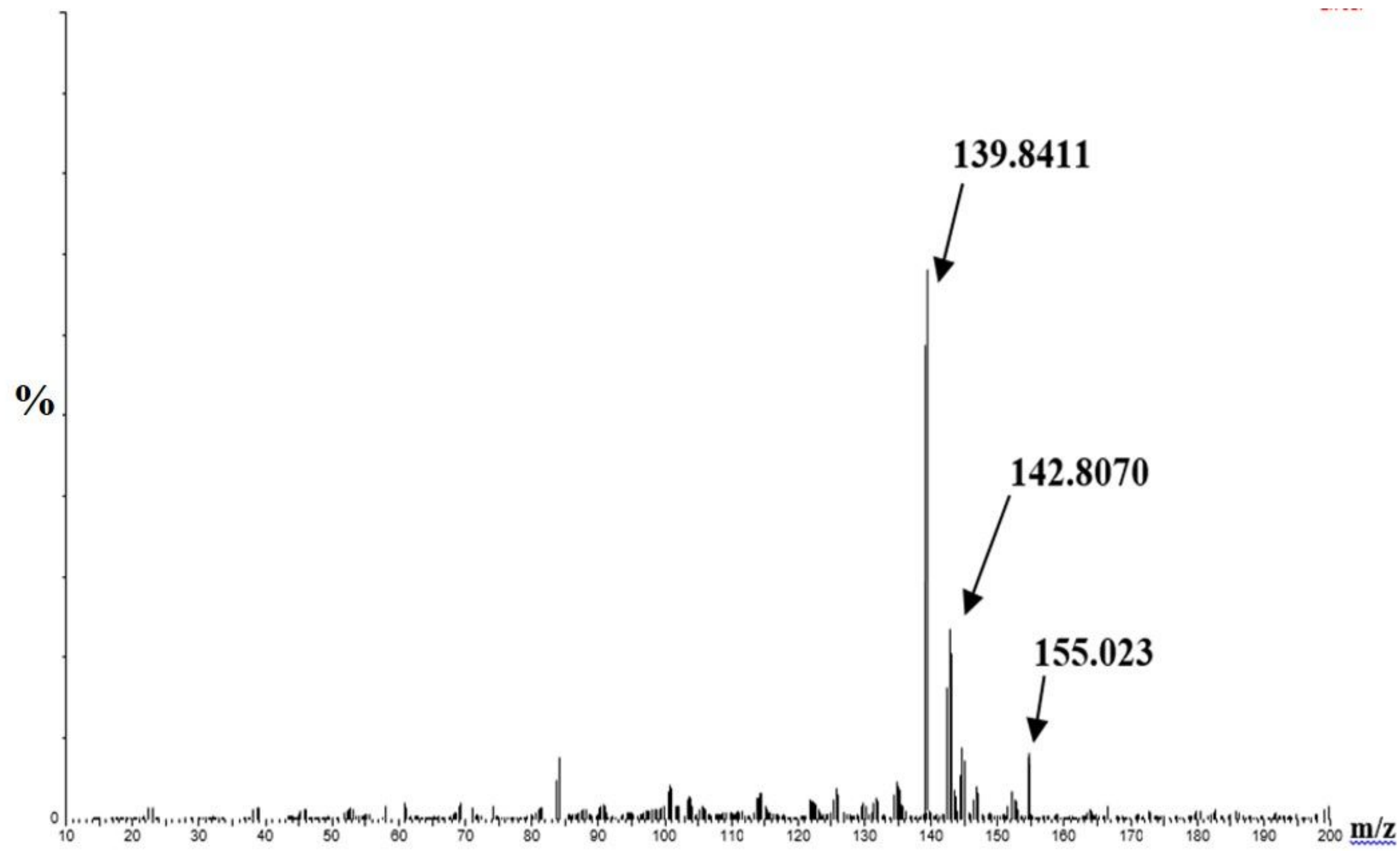

Fig. S15: LC-MS spectra of 2-NP degradation after 15 min

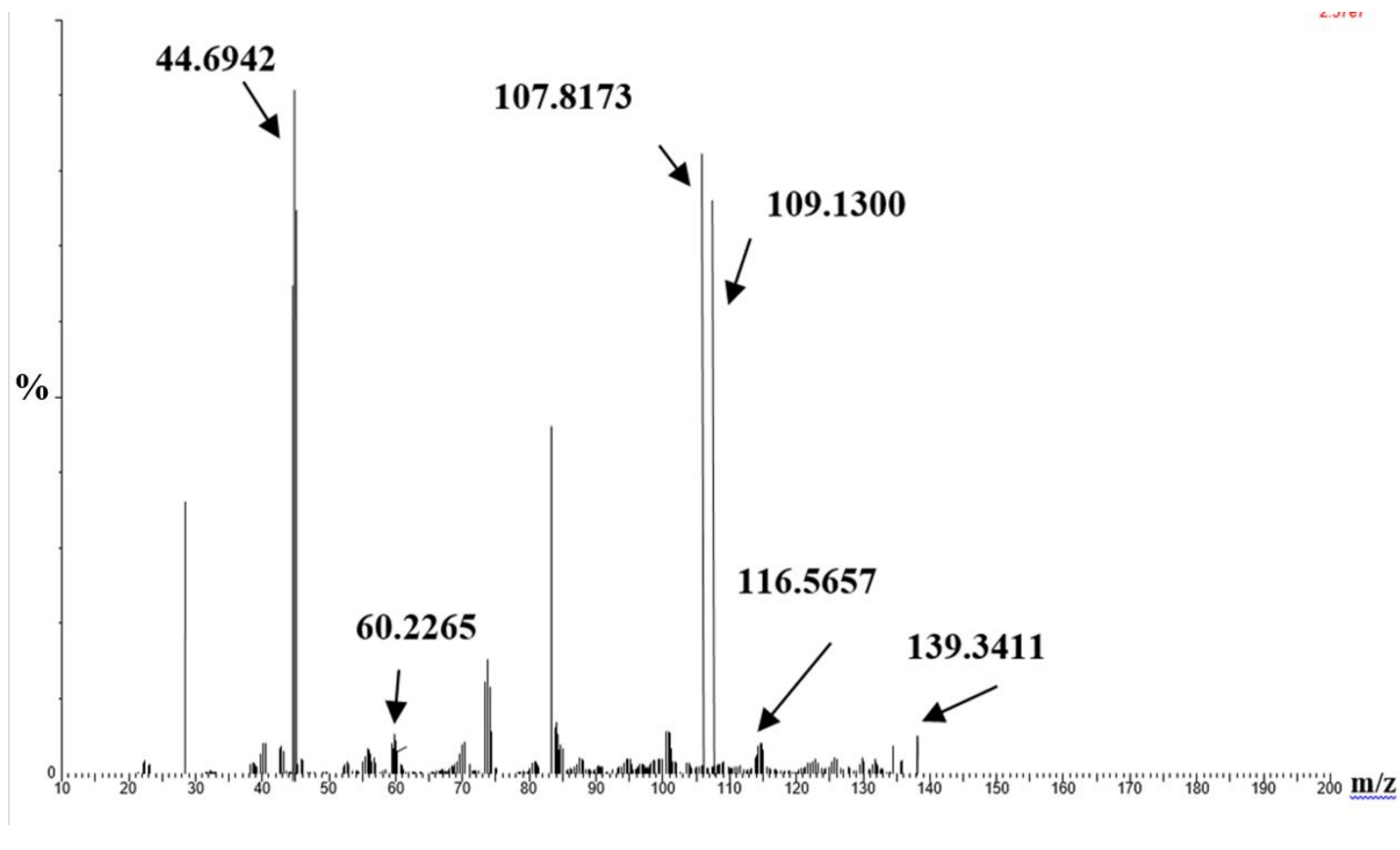

Fig. S16: LC-MS spectra of 2-NP degradation after 60 min

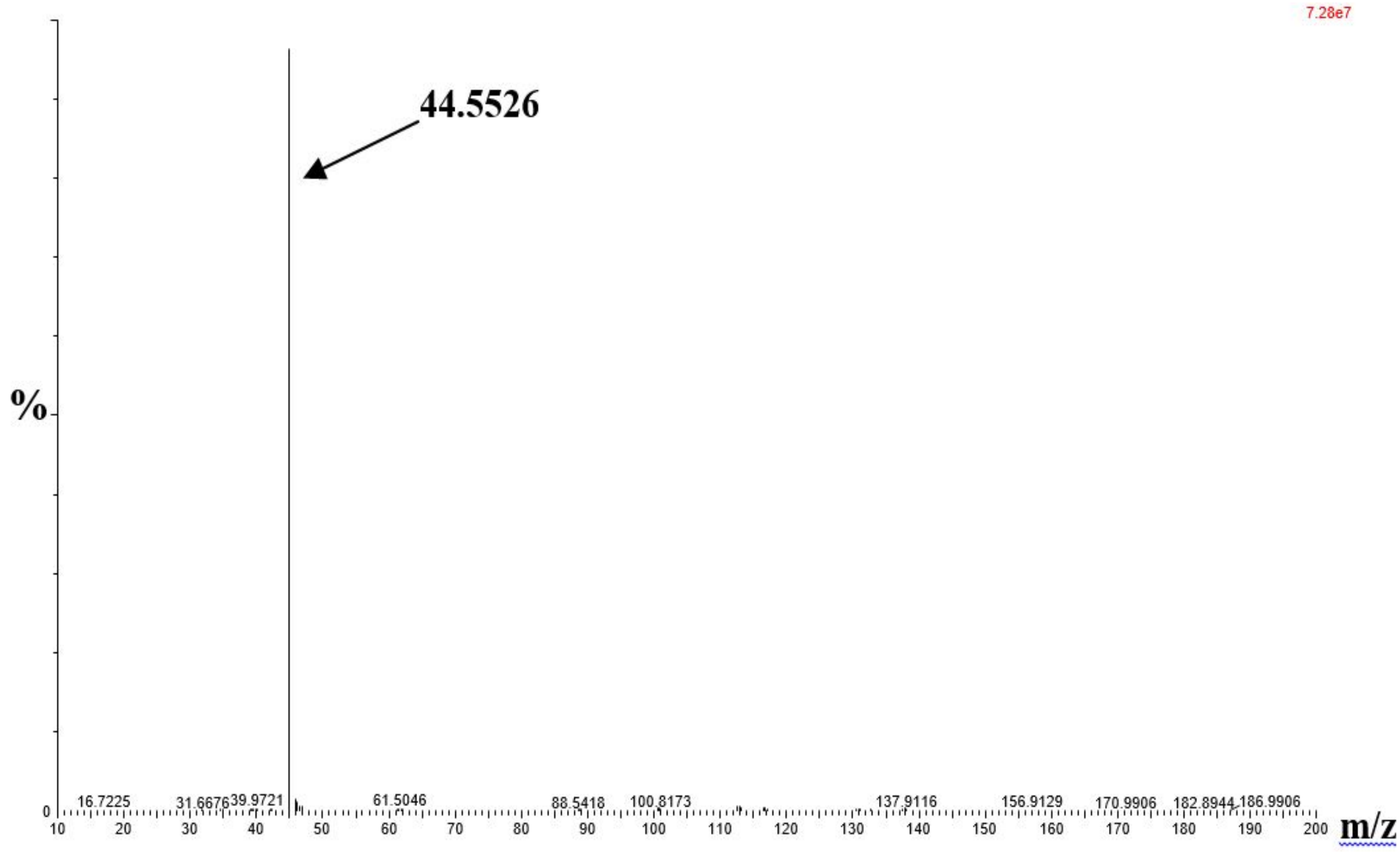

Fig. S17: LC-MS spectra of 2-NP degradation after 160 min

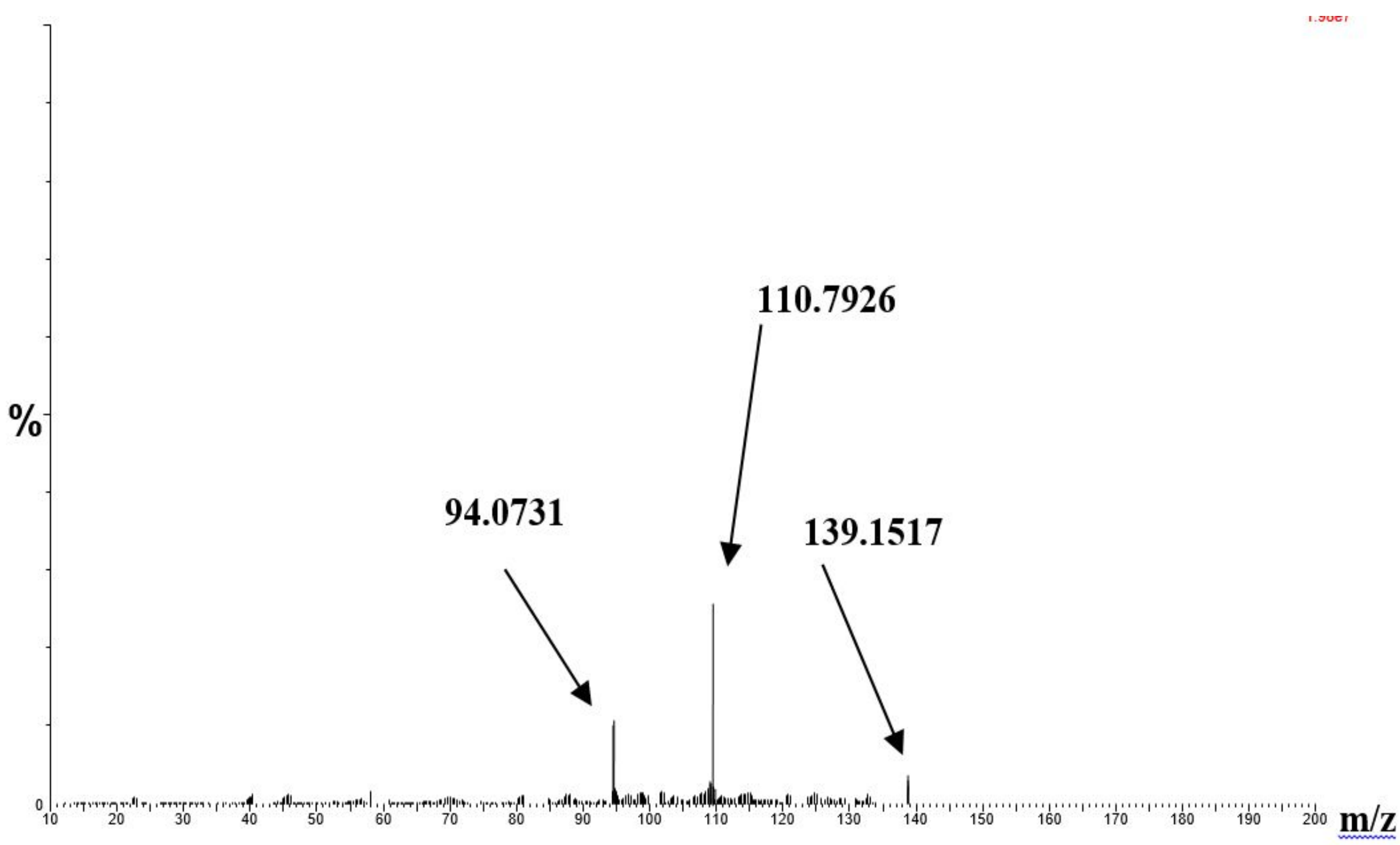

Fig. S18: LC-MS spectra of 4-NP degradation after 15 min

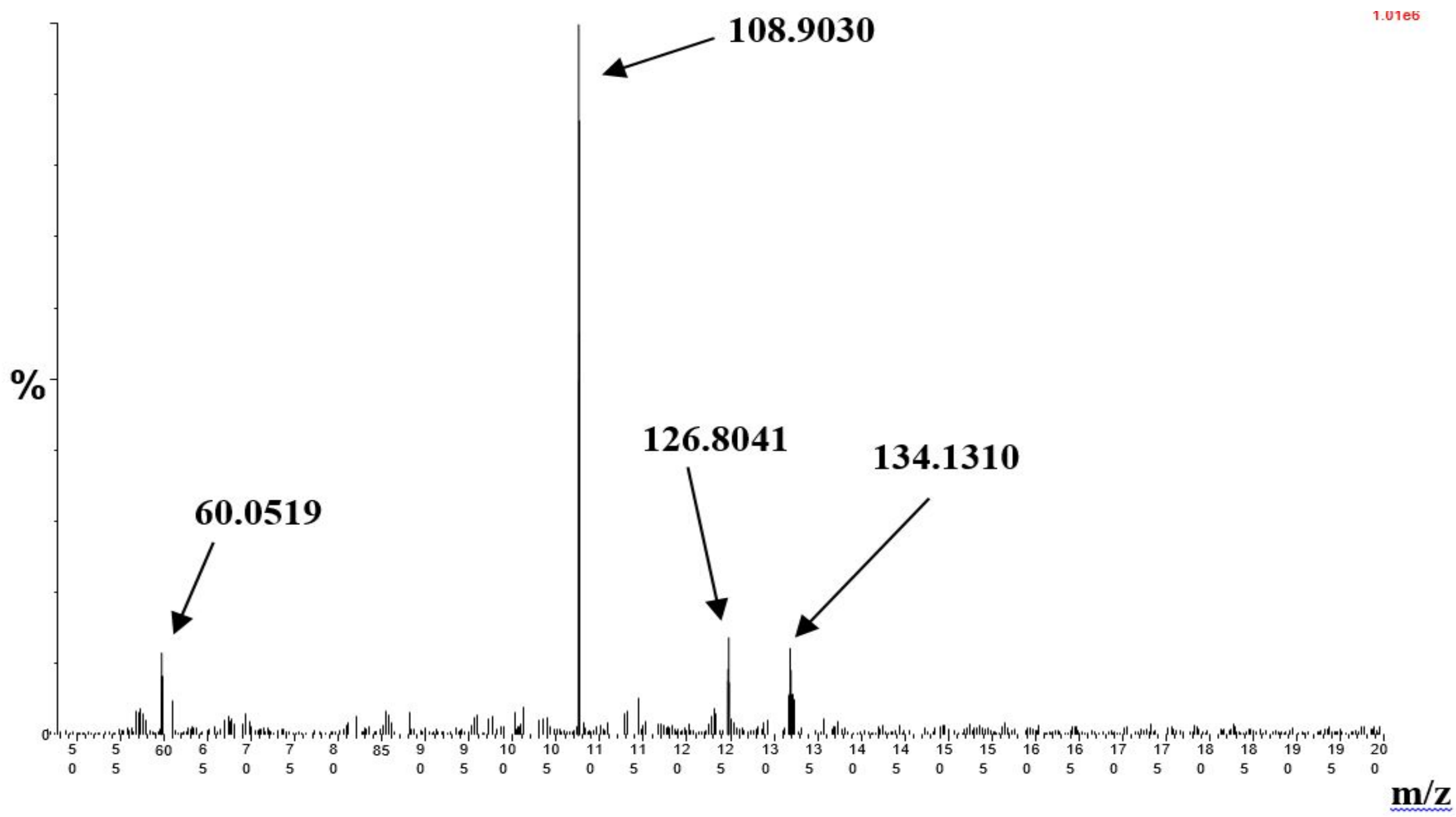

Fig. S19: LC-MS spectra of 4-NP degradation after 60 min

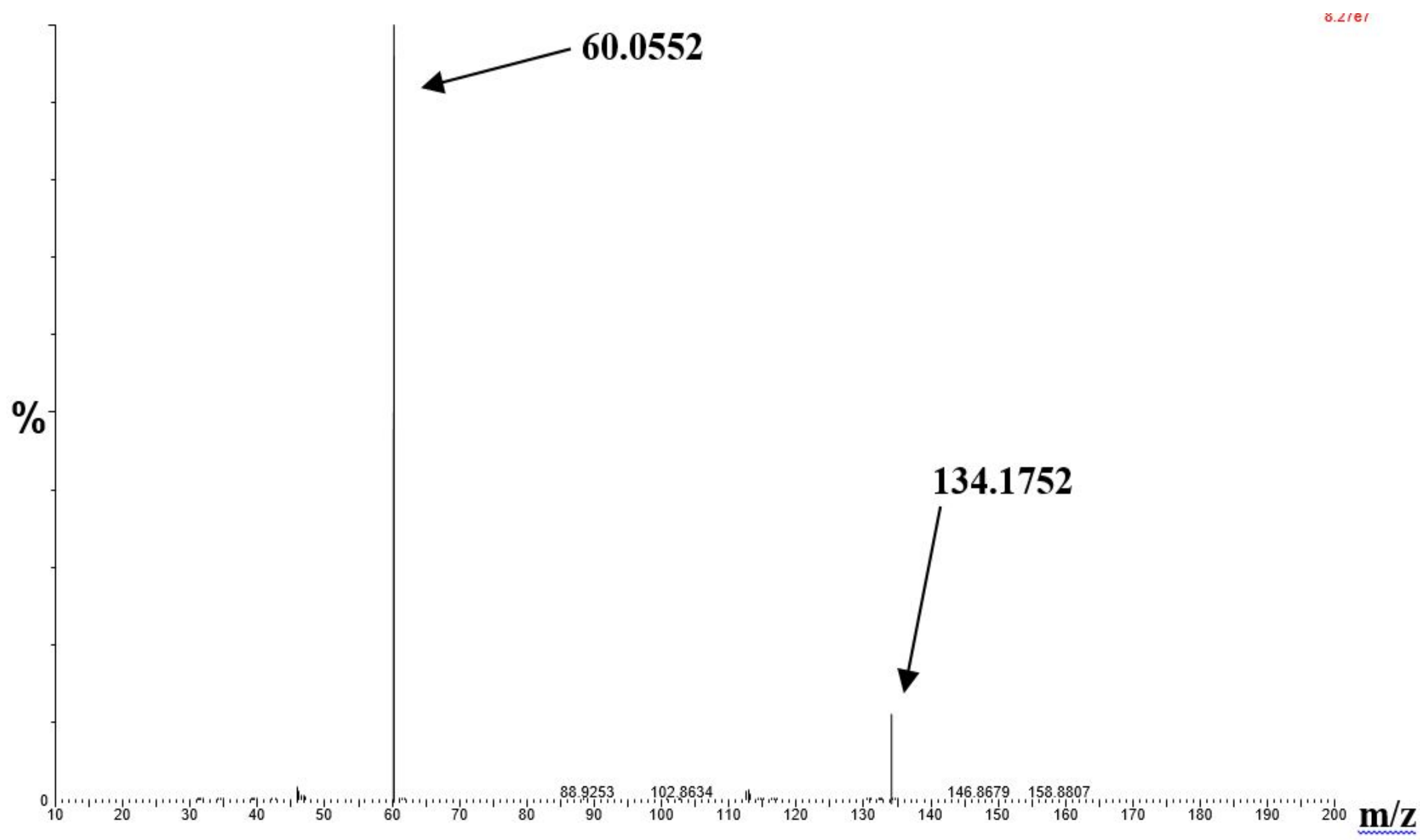

Fig. S20: LC-MS spectra of 4-NP degradation after 160 min

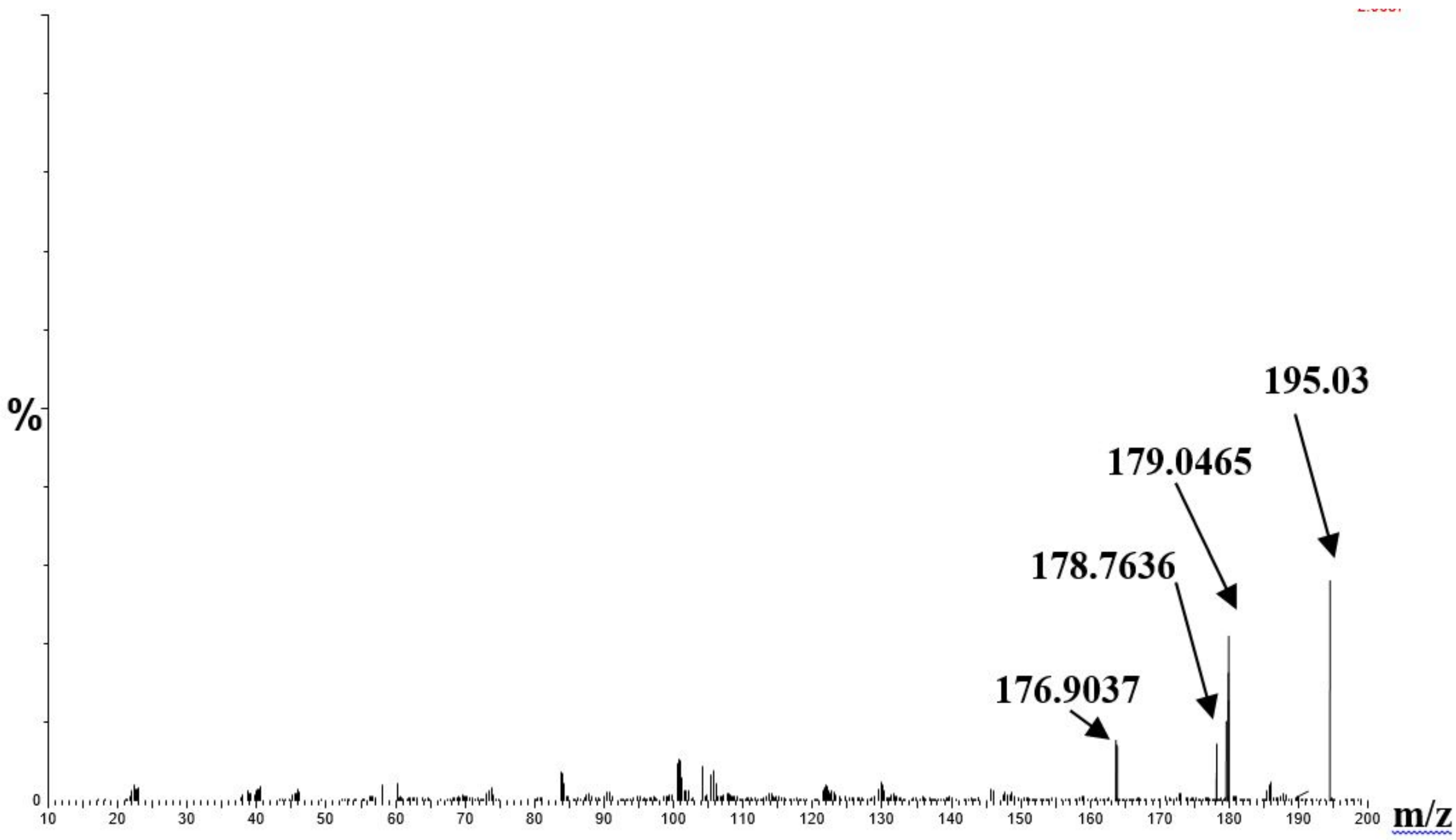

Fig. S21: LC-MS spectra of TCP degradation after 60 min

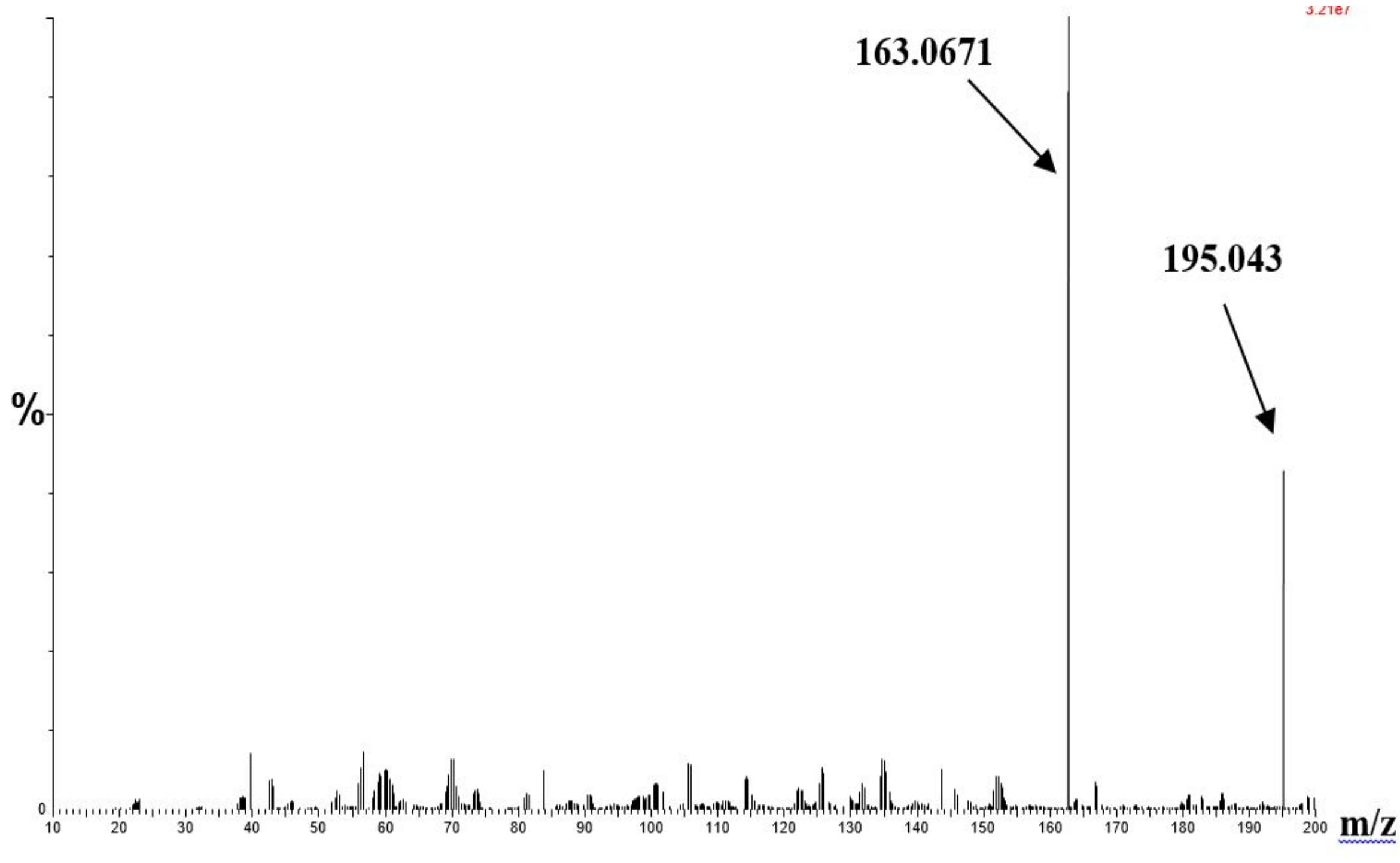

Fig. S22: LC-MS spectra of TCP degradation after 160 min

Table S1: Crystal size of each catalyst sample calculated from XRD data

Sl No.	Sample name	Crystal size (nm)
1	Pure GCN	20.61
2	Pure CF	15.19
3	10-GCN/CF	21.07
4	20-GCN/CF	21.14
5	30-GCN/CF	21.27
6	40-GCN/CF	21.29
7	50-GCN/CF	21.37

Table S2: Elemental analysis of 40-GCN/CF (%)

Sample	C (%)	N (%)	Ca (%)	F (%)
**GCN**	37.81	62	-	-
**CF**	-	-	47.93	52.07
**40-GCN/CF**	33.17	41.85	10.94	14

Table S3: Band positions of GCN and CF sample:

Sl No.	Catalyst	Band gap (E_g)	Conduction band (eV)	Valance band position (eV)
**1**	GCN	2.62	−1.489	1.131
**2**	CF	2.56	− 0.169	2.391

Table S4: Calculated rate constant values for different catalyst samples in different pollutants medium

Catalyst	Rate constant [min^{-1}] (in 2-NP solution)	Rate constant [min^{-1}] (in 4-NP solution)	Rate constant [min^{-1}] (in TCP solution)
No catalyst	0.00211	0.00137	0.0013
GCN	0.00427	0.00276	0.0038
CF	0.00486	0.00314	0.00598
10-GCN/CF	0.00563	0.00495	0.00771
20-GCN/CF	0.00823	0.00723	0.01059
30-GCN/CF	0.00943	0.01004	0.01545
40-GCN/CF	0.01256	0.01399	0.01733

Table S5: Hoogly river water initial and after-treatment composition

Parameters	Initial composition	Final composition (after 3 hours of degradation with 40-GCN/CF
pH	10.2	8.9
Turbidity	68.2 NTU	29.2 NTU
Conductivity	8213.5 ± 20.2 µS/cm	17521.9 ± 15.8 µS/cm
Total dissolved solid (TDS)	238 mg/L	169 mg/L
Total organic carbon (TOC)	4.71 mg/L	2.09 mg/L

Table S 6a: Phytotoxicity assessment in 2-NP solution

[2-NP concentration = 15 ppm, 40-GCN/CF catalyst loading = 50 mg/L]

Seed parameter	Control	DI water		Tap water		Pond water	
		Before photocatalysis	After photocatalysis	Before photocatalysis	After photocatalysis	Before photocatalysis	After photocatalysis
Germination (%)	100	56.18	91.07	46.57	87.29	45.62	85.73
Seed fresh weight (g)	0.431	0.158	0.376	0.107	0.3108	0.087	0.279
Seed dry weight (g)	0.0951	0.0682	0.094	0.061	0.094	0.592	0.091
Root growth (cm)	2.8	1.07	2.1	0.94	2.0	0.9	1.9

Table S6b: Phytotoxicity assessment in 4-NP solution

[4-NP concentration = 15 ppm, 40-GCN/CF catalyst loading = 50 mg/L]

Seed parameter	Control	DI water		Tap water		Pond water	
		Before photocatalysis	After photocatalysis	Before photocatalysis	After photocatalysis	Before photocatalysis	After photocatalysis
Germination (%)	100	55.97	90.1	43.14	84.79	42.09	85.16
Seed fresh weight (g)	0.431	0.148	0.361	0.101	0.306	0.085	0.262
Seed dry weight (g)	0.0951	0.0652	0.093	0.059	0.093	0.56	0.090
Root growth (cm)	2.8	1.06	2.1	0.93	2.0	0.9	1.9

Table S6c: Phytotoxicity assessment in TCP solution

[TCP concentration = 15 ppm, 40-GCN/CF catalyst loading = 58 mg/L]

Seed parameter	Control	DI water		Tap water		Pond water	
		Before photocatalysis	After photocatalysis	Before photocatalysis	After photocatalysis	Before photocatalysis	After photocatalysis
Germination (%)	100	51.18	90.16	41.72	83.85	42.01	82.16
Seed fresh weight (g)	0.431	0.136	0.319	0.1	0.301	0.082	0.247
Seed dry weight (g)	0.0951	0.0617	0.091	0.057	0.09	0.54	0.089
Root growth (cm)	2.8	1.06	2.1	0.91	1.8	0.9	1.7

Table S7: Possible intermediates of 2-NP degradation identified in LC-MS analysis

Sl No.	Possible intermediate	Chemical structure	m/z
1	2-nitrobenzene-1,4-diol	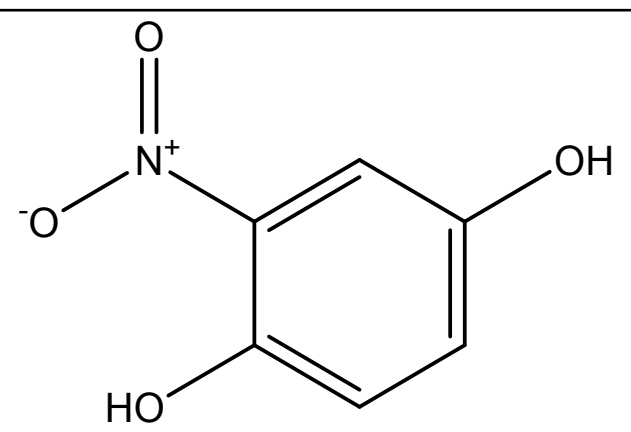	155
2	(2Z,4Z)-hexa-2,4-dienedioic acid	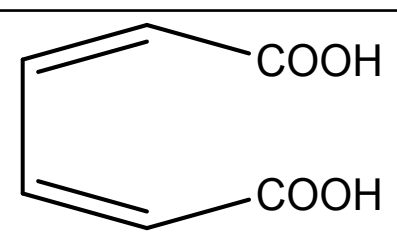	142
3	Maleic acid	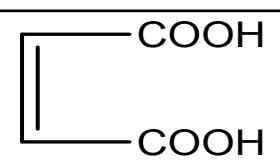	116
4	Hydroquinone	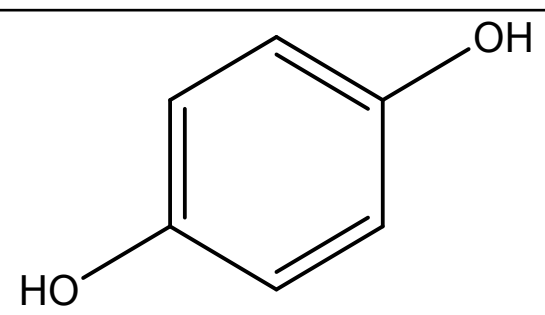	109
5	Benzoquinone	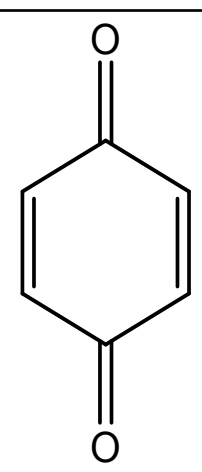	107
6	Acetic acid	CH_3COOH	60
7	Acetaldehyde	CH_3CHO	44

Table S8: Possible intermediates of 4-NP degradation identified in LC-MS analysis

Sl No.	Possible intermediate	Chemical structure	m/z
1	But-2-enedioic acid	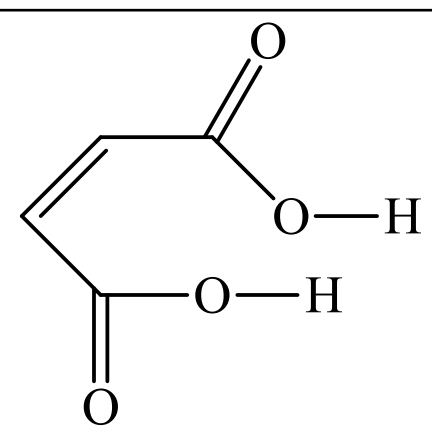	134.08
2	Benzene-1,2,4-triol	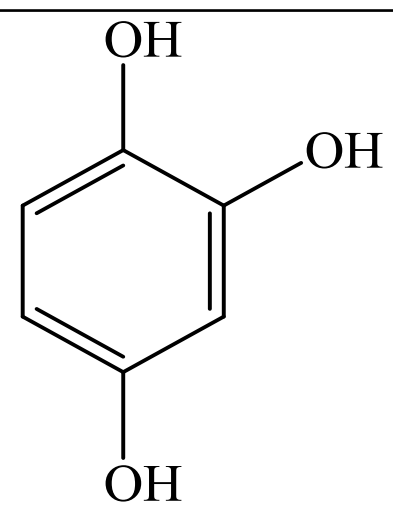	126.11
3	Hydroquinone	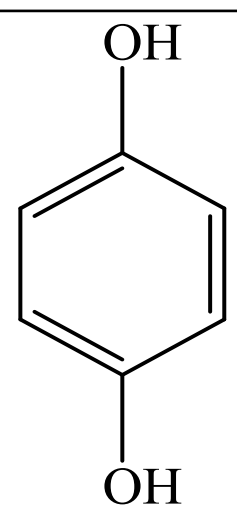	110.11
4	Phenol	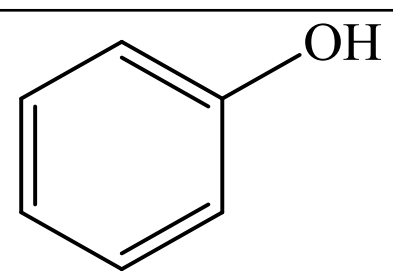	94.11
5	Acetic acid	CH_3COOH	60

Table S9: Possible intermediates of TCP degradation identified in LC-MS analysis

Sl No.	Possible intermediate	Chemical structure	m/z
1	2,6-dichlorophenol	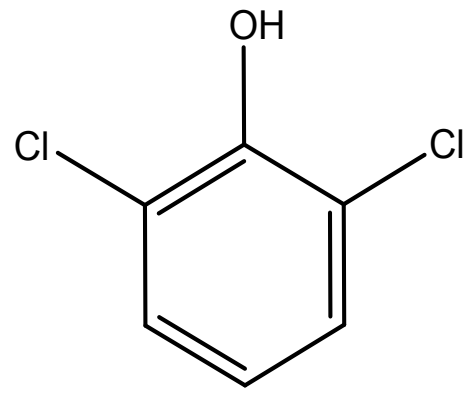	163
2	2,6-dichlorobenzene-1,4-diol	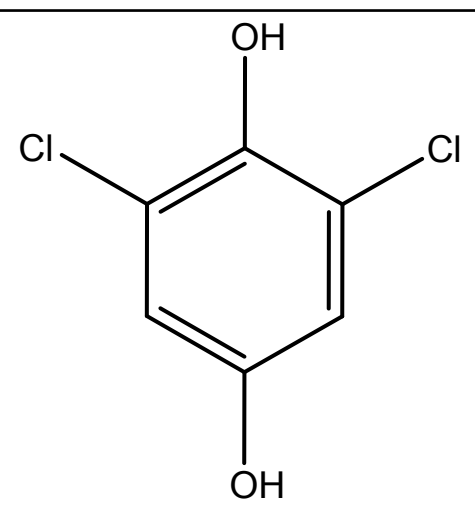	179.005
3	2,6-dichloro-4-hydroxycyclohexa-2,5-dienone	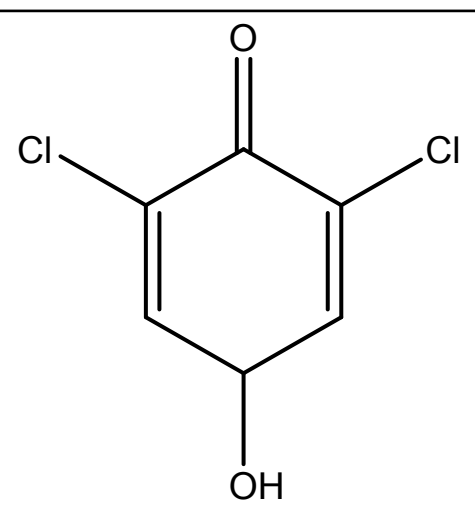	178.9
4	2,6-dichlorocyclohexa-2,5-diene-1,4-dione	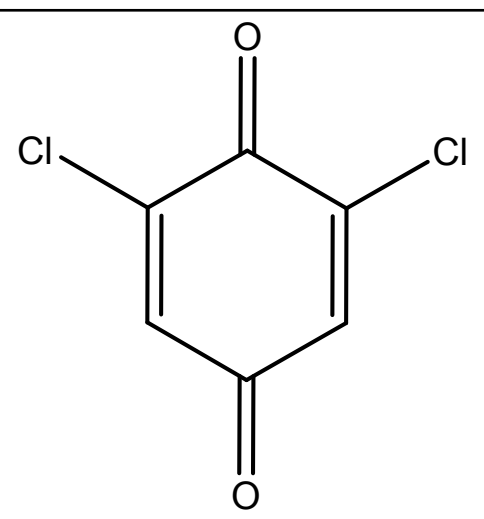	176.94
5	2-hydroxypentanedioic acid	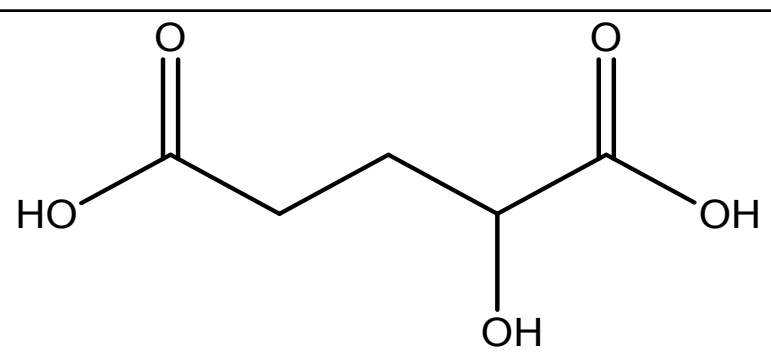	119.0267
6	Maleic acid	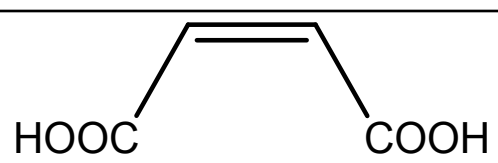	115
7	(E)-but-2-enoic acid	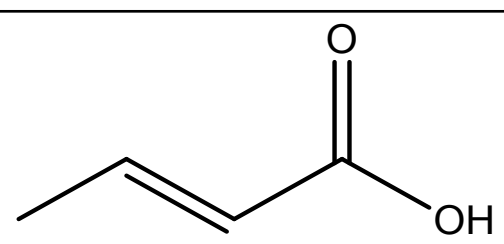	85
8	Acetic acid	CH3COOH	60

Table S 10: Different organic pollutants degradation efficiency of previously reported $g-C_3N_4$ based photocatalyst in visible light

Photocatalyst	Synthesis method	Degradation percentage in visible light	Reference
NiO-loaded $g-C_3N_4$ heterojunction photocatalyst	Ultrasonic-assisted method	80 % of 4-NP was photodegraded under visible light irradiation for 3 h.	M. Ismael et al., 2023
TiO_2/$g-C_3N_4$ heterojunction	Pyrolysis	95.8% degradation for phenol, *p*-Nitrophenol, o-Cresol and p-Cresol within 100 min	X. Qu et al., 2022
$g-C_3N_4$ nanosheets sustained Bi_2S_3 heterojunction	Microwave-assisted fabrication	Reduction of 4-NP to 4-AP was exhibited approximately 100%	Dasari Ayodhya& Guttena Veerabhad ram, 2021
ZIF-67/$g-C_3N_4$ heterojunction	Pyrolysis and coprecipitation followed by continuous stirring for 12 h	97.5% reduction of 4-nitrophenol	D. Roy et al., 2021
$g-C_3N_4$ /WO_3 heterojunction	Thermal-induced self-polymerization method	94.5% removal efficiency of PNP	X. Zhang et al., 2021

	and hydrothermal reaction		
B co-doped g-C_3N_4 nanotube@MnO_2 heterojunction	Pyrolysis method followed by hydrothermally heating at 160 °C for 6 h	Photodegradation efficiency of 92 – 99 % is achieved after 15 min of irradiation.	M. Dung Nguyen et al., 2023
Tubular g-C_3N_4 with Bi_2O_3 nanosheets heterojunction	Hydrothermal assisted calcination and composites were prepared by the solvothermal method	The removal efficiency of TC reached 94% in 100 min, and that of RhB reached 99% in 60 min	X. Pei, W. An, H. Zhao et al., 2023
3D spherical CuO@g-C_3N_4 composites	Rapid thermal polymerization followed by urea hydrothermal method	The removal rate of 2,4,6,-TCP reached 94 % at 25 min	S. Wang et al., 2024
g-C_3N_4/MoO_3-x heterostructures	Two-step thermal polymerization route	The phenol removal efficiency reached 98% within 60 min	Q. Che et al., 2023

Reference:

1. Ayodhya, D., & Veerabhadram, G. (2021). Microwave-assisted fabrication of g-C_3N_4 nanosheets sustained Bi_2S_3 heterojunction composites for the catalytic reduction of 4-nitrophenol. *Environmental Technology*, *42*(6), 826-841.
2. Ismael, M. (2023). Facile synthesis of NiO-loaded g-C_3N_4 heterojunction photocatalyst for efficient photocatalytic degradation of 4-nitrophenol under visible light irradiation. *Journal of Photochemistry and Photobiology A: Chemistry*, *439*, 114576.
3. Nguyen, M. D., Nguyen, T. B., Tran, L. H., Nguyen, T. G., Fatimah, I., Kuncoro, E. P., & Doong, R. A. (2023). Z-scheme S, B co-doped g-C_3N_4 nanotube@ MnO_2 heterojunction with visible-light-responsive for enhanced photodegradation of diclofenac by peroxymonosulfate activation. *Chemical Engineering Journal*, *452*, 139249.
4. Pei, X., An, W., Zhao, H., He, H., Fu, Y., & Shen, X. (2023). Enhancing visible-light degradation performance of g-C_3N_4 on organic pollutants by constructing heterojunctions via combining tubular g-C_3N_4 with Bi_2O_3 nanosheets. *Journal of Alloys and Compounds*, *934*, 167928.
5. Qu, X., Chen, C., Lin, J., Qiang, W., Zhang, L., & Sun, D. (2022). Engineered defect-rich TiO_2/g-C_3N_4 heterojunction: A visible-light-driven photocatalyst for efficient degradation of phenolic wastewater. *Chemosphere*, *286*, 131696.
6. Roy, D., Neogi, S., & De, S. (2021). Highly efficient reduction of p-Nitrophenol by sodium borohydride over binary ZIF-67/g-C_3N_4 heterojunction catalyst. *Journal of Environmental Chemical Engineering*, *9*(6), 106677.
7. Song, T., Xie, C., Che, Q., & Yang, P. (2023). Enhanced carrier separation in g-C_3N_4/MoO_3x heterostructures towards efficient phenol removal. *Journal of Industrial and Engineering Chemistry*, *122*, 415-425.

8. Wang, S., Yuan, C., Chen, W., Niu, Y., Yan, Y., Li, F., & Jiang, H. (2024). 3D spherical CuO@ g-C_3N_4 composites activating peroxymonosulfate for high efficient degradation of 2, 4, 6-trichlorophenol: The mechanism of $^1O_2$ generation. *Chemical Engineering Journal*, *480*, 148050.
9. Zhang, X., Wang, X., Meng, J., Liu, Y., Ren, M., Guo, Y., & Yang, Y. (2021). Robust Z-scheme g-C_3N_4/WO_3 heterojunction photocatalysts with morphology control of WO_3 for efficient degradation of phenolic pollutants. *Separation and Purification Technology*, *255*, 117693.
10. Zhang, Z., Feng, C., Liu, C. *et al.* Electrochemical deposition as a universal route for fabricating single-atom catalysts. *Nat Commun* 11, 1215 (2020)
